# Osteopathology in Rhinocerotidae from 50 Million Years to the Present

**DOI:** 10.1371/journal.pone.0146221

**Published:** 2016-02-03

**Authors:** Kelsey T. Stilson, Samantha S. B. Hopkins, Edward Byrd Davis

**Affiliations:** 1 Jackson School of Geosciences, University of Texas, Austin, TX, 78712–1692, United States of America; 2 Department of Geological Sciences, University of Oregon, Eugene, OR, 97403–1272, United States of America; 3 Clark Honors College, University of Oregon, Eugene, OR, 97403–1293, United States of America; 4 Museum of Natural and Cultural History, University of Oregon, Eugene, OR, 97403–1224, United States of America; NYIT College of Osteopathic Medicine, UNITED STATES

## Abstract

Individual elements of many extinct and extant North American rhinocerotids display osteopathologies, particularly exostoses, abnormal textures, and joint margin porosity, that are commonly associated with localized bone trauma. When we evaluated six extinct rhinocerotid species spanning 50 million years (Ma), we found the incidence of osteopathology increases from 28% of all elements of Eocene *Hyrachyus eximius* to 65–80% of all elements in more derived species. The only extant species in this study, *Diceros bicornis*, displayed less osteopathologies (50%) than the more derived extinct taxa. To get a finer-grained picture, we scored each fossil for seven pathological indicators on a scale of 1–4. We estimated the average mass of each taxon using M1-3 length and compared mass to average pathological score for each category. We found that with increasing mass, osteopathology also significantly increases. We then ran a phylogenetically-controlled regression analysis using a time-calibrated phylogeny of our study taxa. Mass estimates were found to significantly covary with abnormal foramen shape and abnormal bone textures. This pattern in osteopathological expression may reflect a part of the complex system of adaptations in the Rhinocerotidae over millions of years, where increased mass, cursoriality, and/or increased life span are selected for, to the *detriment* of long-term bone health. This work has important implications for the future health of hoofed animals and humans alike.

## Introduction

Rhinos diverged from their closest living relative, the tapir, about 50.3 million years ago (Ma) [1,2,3] and quickly increased in abundance and species richness through the mid-Cenozoic. The rhinocerotid lineage is hypothesized to have diversified into four major clades in North America and Eurasia: the Diceratheriinae in the Oligocene, and the Aceratheriinae, Teleoceratinae, and Rhinocerotinae in the Miocene [4, 5]. Cursoriality, or the habit of running, has been hypothesized to have been maintained through the majority of these lineages [5,6]. These grazing and browsing lineages were some of the most numerous and widespread large mammals in the mid-Cenozoic, with frequent periods of migration between North America, Eurasia, and Africa [7, 8, 9]. About five million years ago the last North American genus, *Teleoceras*, disappeared from the fossil record [5]. North American rhino populations are estimated to have been smaller in size and weight than their Eurasian relatives [10], but both continents record taxa with increasingly robust and graviportal skeletons over time [5,11].

There are five extant species of rhino, all within the Rhinocerotinae. Two species are in Africa and three in Asia. As with many Animalia, a certain percentage of extinct and extant taxa show evidence of bony and soft-tissue pathologies [12, 13,14], with a recent increase in studies of pathologies in captive modern populations and individuals as wild numbers decline. For Rhinocerotidae, the great majority of studies are reports on captive rhinos with foot disorders [4,12,15,16], discovered during surgery or necropsy. For example, a recent study examined 27 modern captive individuals and a subset were scanned using computed tomography [13]. They found the majority displayed osteoarthritis and/or enthesopathy, particularly in the feet, and hypothesized that increased stress or strain, nutritional imbalance, habitat, and/or ascending infection could contribute the observed pathologies. A similar study [14] noted a wide range of osteopathologies in *Ceratotherium simum* and *Rhinoceros unicornis* using computed tomography and hypothesized that care, the weight of the animal, nutrition, or age could all contribute to pathological expression [14]. Extinct taxa have also displayed arthritis-like features, the most prominent being an increase in the frequency of spondylarthropathy (inflammatory arthritis, indicated by abnormal joint erosion or bone fusion) from around 10% in Oligocene Equidae and Rhinocerotidae to around 30% in the Holocene [17]. The common thread from these studies is the type of pathology recorded. These pathologies could all be grouped not as sudden traumatic events but, like runner’s knee or tennis elbow, growth or destruction of bone in response to increased physical stress over the lifetime of an individual.

Bone growth in mammals is promoted by a combination of mechanical (low level stress) and hormone stimulation [18]. After primary growth and development of a mammal is complete, bone repair and remodeling responds primarily to local stimulation [16,18,19] caused by mechanical load. Local osteocytes (bone cells) respond to bone damage and wear with cell hyperplasia (increased cell growth or proliferation). Extensive cellular damage, localized biomechanics (e.g. joint loading, genetic predisposition, and the environment are all potential causal factors of bone degeneration, inflammation and infection in the bone or surrounding tissue [12, 16, 20]. Thus, continuous remodeling of bone can result in bone morphologies and pathologies that reflect what happened to the bone when it was part of a living organism.

Increased mechanical load increases the likelihood of arthropathies such as proliferative joint diseases, erosive joint diseases, synovitis, and traumatic injury [16]. We will briefly examine the major arthropathies, but emphasize that the goal of this paper is not to diagnose the Rhinocerotidae lineage with a specific disease, but record and examine the osteopathologies that are possibly the result of these or related diseases. Four indicators of osteoarthritis (i.e. proliferative joint disease) commonly used in anthropologic studies are: eburnation, a wearing away of the bony articular surface, marginal osteophytes (known as lipping), sclerotic lesions or pitting on the articular surfaces, and alteration in the shape of the joint [21,22]. There are many other erosive arthropathies, but the most characteristic is rheumatoid arthritis (RA). RA includes symmetrical erosions of the hands and feet, minimal new bone formation, erosions, and osteoporosis [16]. Synovitis includes cortical erosion and irregular cysting [16]. Cysting, and ankyloses, or the fusion of a joint [16, 22] may also result from increased mechanical load. Other pathologies related to mechanical stress include inflammation of the periosteum, which can form exostoses. Traumatic breaking and healing of the bone may occur, in conjunction with chronic arthropathies.

We initially expected to see a correlation between the severity of pathological expression and an increase in rhino mass and cursorial habits, because of the known correlation between osteopathology and mechanical stress [16, 18, 20, 21, 22], as well as previous observations of pathologies in rhinos [4, 13,14,15]. We reasoned that an increase in mass would put greater stress on bones and joints, increasing the likelihood that arthritis-like pathologies, such as osteophyte formation and articular surface degradation, would occur. If this were the case, the tendency to develop stress-related osteopathologies would be trackable and predictable. We asked two overarching questions in this study: (1) Do these osteopathologic features exhibit a trend over time? And (2) what is the relationship between mass and osteopathology?

## Materials and Methods

To determine the relationship between mass and osteopathology through time, we collected data on osteopathologies from a number of extinct and extant taxa in the family Rhinocerotidae, Table 1, and an outgroup, *Hyrachyus eximius*, a perissodactyl sister group to the Rhinocerotidae [8, 23]. We collected data from localities with a large number of rhino skeletal elements to avoid individual preservation bias as much as possible, forming a series of species-level “snapshots” of the rhino lineage. Fossil species were chosen to span the temporal range of rhinocerotids and for the presence of adequate samples of identified elements, Table 2. Data resolution is not on the order of populations, but species in formations; this lumping of occurrences allows us to achieve a statistically adequate sample. For example, there are 15 different localities that comprise the *Hyrachyus eximius* sample, but they are all part of the Bridger Formation within Uinta County, Wyoming. No permits were required for the described study, which complied with all relevant regulations.

**Table 1:** Summary of species in this study.

Name	Age Range (MA)	Mass (kg)	NISP	MNI	Formation
***Hyrachyus eximius***^***†***^	50.5–46.2	36.3	275	116	Bridger
***Trigonias osborni***^***†***^	37.2–33.9	677	115	34	White River
***Menoceras arikarense***^***†***^	24.8–20.4	375	83	61	Harrison
***Diceratherium niobrarense***^***†***^	33.3–30.8	1010	72	26	John Day
***Aphelops mutilis***^***†***^	10.3–4.9	1840	110	53	Ogalla Group
***Teleoceras hicksi***^***†***^	10.3–4.9	1660	65	29	Shutler/ Mascall/ Rattlesnake
***Diceros bicornis***	0	1080	75	4	——

Summary of species in this study and related age ranges, mass, number of identified specimens (NISP), minimum number of individuals (MNI), and the geological formation the fossils are associated with. Data compiled from Fortelius and Kappelman [24], Cerdeño [8, 10], Prothero [5], Mendoza [25], and Owen-Smith [26]. Mass Estimate M1-3 Length from Radinsky 1967 [23] for *H*. *eximius* and Prothero [5] for the rest.

**Table 2:** Numbers and Localities of all Specimens Used in This Study.

Prefix	Specimen Num.	Genus	Species	element	NISP	Locality Name	Formation	Age
AMNH	1645	*Hyrachyus*^***†***^	*eximius*^***†***^	pelvis	1	Twin Buttes	Bridger	Bridgerian
AMNH	11652	*Hyrachyus*^***†***^	*eximius*^***†***^	pelvis	3	Little Dry Cr'k	Bridger	Bridgerian
AMNH	12364	*Hyrachyus*^***†***^	*eximius*^***†***^	pelvis	2	30 ft. above upper white stratus	Bridger	Bridgerian
AMNH	1621	*Hyrachyus*^***†***^	*eximius*^***†***^	femur	1	Bridger Basin	Bridger	Bridgerian
AMNH	1621	*Hyrachyus*^***†***^	*eximius*^***†***^	tibia	1	Bridger Basin	Bridger	Bridgerian
AMNH	1623	*Hyrachyus*^***†***^	*eximius*^***†***^	radius	1	Bridger Basin	Bridger	Bridgerian
AMNH	1638	*Hyrachyus*^***†***^	*eximius*^***†***^	radius	1	Cottonwood Corral	Bridger	Bridgerian
AMNH	1638	*Hyrachyus*^***†***^	*eximius*^***†***^	ulna	1	Cottonwood Corral	Bridger	Bridgerian
AMNH	1640	*Hyrachyus*^***†***^	*eximius*^***†***^	femur	1	Bridger Basin	Bridger	Bridgerian
AMNH	1640	*Hyrachyus*^***†***^	*eximius*^***†***^	radius	1	Bridger Basin	Bridger	Bridgerian
AMNH	1641	*Hyrachyus*^***†***^	*eximius*^***†***^	femur	1	Bridger Basin	Bridger	Bridgerian
AMNH	1644	*Hyrachyus*^***†***^	*eximius*^***†***^	tibia	1	Bridger Basin	Bridger	Bridgerian
AMNH	1645	*Hyrachyus*^***†***^	*eximius*^***†***^	tibia	1	Twin Buttes	Bridger	Bridgerian
AMNH	1646	*Hyrachyus*^***†***^	*eximius*^***†***^	humerus	1	Twin Buttes	Bridger	Bridgerian
AMNH	1646	*Hyrachyus*^***†***^	*eximius*^***†***^	radius (juvenile)	1	Twin Buttes	Bridger	Bridgerian
AMNH	1646	*Hyrachyus*^***†***^	*eximius*^***†***^	tibia	1	Twin Buttes	Bridger	Bridgerian
AMNH	1646	*Hyrachyus*^***†***^	*eximius*^***†***^	ulna	1	Twin Buttes	Bridger	Bridgerian
AMNH	1903	*Hyrachyus*^***†***^	*eximius*^***†***^	ulna	1	Henry's Fork LT	Bridger	Bridgerian
AMNH	11693	*Hyrachyus*^***†***^	*eximius*^***†***^	femur head	1	Bridger	Bridger	Bridgerian
AMNH	11693	*Hyrachyus*^***†***^	*eximius*^***†***^	radius—broken into two parts	1	Bridger	Bridger	Bridgerian
AMNH	11693	*Hyrachyus*^***†***^	*eximius*^***†***^	tibia	1	Bridger	Bridger	Bridgerian
AMNH	11707	*Hyrachyus*^***†***^	*eximius*^***†***^	femur	1	Henry's Fork LT	Bridger	Bridgerian
AMNH	11712	*Hyrachyus*^***†***^	*eximius*^***†***^	femur	1	Cat-tail Spring	Bridger	Bridgerian
AMNH	11712	*Hyrachyus*^***†***^	*eximius*^***†***^	tibia	1	Church Buttes	Bridger	Bridgerian
AMNH	12179	*Hyrachyus*^***†***^	*eximius*^***†***^	femur	1	Bridger	Bridger	Bridgerian
AMNH	12179	*Hyrachyus*^***†***^	*eximius*^***†***^	tibia- lower	1	Bridger	Bridger	Bridgerian
AMNH	12179	*Hyrachyus*^***†***^	*eximius*^***†***^	tibia- upper	1	Bridger	Bridger	Bridgerian
AMNH	12225	*Hyrachyus*^***†***^	*eximius*^***†***^	humerus	1	Summer's Dry Cr'k	Bridger	Bridgerian
AMNH	12225	*Hyrachyus*^***†***^	*eximius*^***†***^	radius	1	Summer's Dry Cr'k	Bridger	Bridgerian
AMNH	12225	*Hyrachyus*^***†***^	*eximius*^***†***^	ulna	1	Summer's Dry Cr'k	Bridger	Bridgerian
AMNH	12356	*Hyrachyus*^***†***^	*eximius*^***†***^	radius, ulna, distal humerus (articulated)	1	Mouth of Summer's Dry Creek	Bridger	Bridgerian
AMNH	12364	*Hyrachyus*^***†***^	*eximius*^***†***^	distal tibia	1	Henry's Fork	Bridger	Bridgerian
AMNH	12364	*Hyrachyus*^***†***^	*eximius*^***†***^	femur	1	Henry's Fork	Bridger	Bridgerian
AMNH	12364	*Hyrachyus*^***†***^	*eximius*^***†***^	fibula	1	Henry's Fork	Bridger	Bridgerian
AMNH	12364	*Hyrachyus*^***†***^	*eximius*^***†***^	tibia	1	Henry's Fork	Bridger	Bridgerian
AMNH	12665	*Hyrachyus*^***†***^	*eximius*^***†***^	femur	1	Grizzly Buttes	Bridger	Bridgerian
AMNH	12665	*Hyrachyus*^***†***^	*eximius*^***†***^	tibia	1	Grizzly Buttes	Bridger	Bridgerian
AMNH	12673	*Hyrachyus*^***†***^	*eximius*^***†***^	radius	1	Henry's Fork LT	Bridger	Bridgerian
AMNH	12673	*Hyrachyus*^***†***^	*eximius*^***†***^	ulna	1	Henry's Fork LT	Bridger	Bridgerian
AMNH	12675	*Hyrachyus*^***†***^	*eximius*^***†***^	femur	1	Black's Fork above Millersville	Bridger	Bridgerian
AMNH	12675	*Hyrachyus*^***†***^	*eximius*^***†***^	femur	1	Black's Fork above Millersville	Bridger	Bridgerian
AMNH	12675	*Hyrachyus*^***†***^	*eximius*^***†***^	fibula	1	Black's Fork above Millersville	Bridger	Bridgerian
AMNH	12675	*Hyrachyus*^***†***^	*eximius*^***†***^	tibia	1	Black's Fork above Millersville	Bridger	Bridgerian
AMNH	93050	*Hyrachyus*^***†***^	*eximius*^***†***^	femur	1	Henry's Fork LT	Bridger	Bridgerian
AMNH	93050	*Hyrachyus*^***†***^	*eximius*^***†***^	radius	1	Henry's Fork LT	Bridger	Bridgerian
AMNH	93050	*Hyrachyus*^***†***^	*eximius*^***†***^	ulna	1	Henry's Fork LT	Bridger	Bridgerian
AMNH	93052	*Hyrachyus*^***†***^	*eximius*^***†***^	femur	1	Grizzly Buttes	Bridger	Bridgerian
AMNH	93058	*Hyrachyus*^***†***^	*eximius*^***†***^	tibia	1	Grizzly Buttes	Bridger	Bridgerian
AMNH	93059	*Hyrachyus*^***†***^	*eximius*^***†***^	tibia	1	Henry's Fork LT	Bridger	Bridgerian
AMNH	93060	*Hyrachyus*^***†***^	*eximius*^***†***^	femur	1	Bridger Basin	Bridger	Bridgerian
AMNH	93064	*Hyrachyus*^***†***^	*eximius*^***†***^	femur	1	Henry's Fork LT	Bridger	Bridgerian
AMNH	93065	*Hyrachyus*^***†***^	*eximius*^***†***^	tibia	1	Grizzly Buttes	Bridger	Bridgerian
AMNH	93066	*Hyrachyus*^***†***^	*eximius*^***†***^	tibia	1	Bridger	Bridger	Bridgerian
AMNH	1644-A	*Hyrachyus*^***†***^	*eximius*^***†***^	femur	1	Bridger Basin	Bridger	Bridgerian
AMNH	5065-A	*Hyrachyus*^***†***^	*eximius*^***†***^	humerus	1	Bridger Basin	Bridger	Bridgerian
AMNH	5065-A	*Hyrachyus*^***†***^	*eximius*^***†***^	ulna	1	Bridger Basin	Bridger	Bridgerian
AMNH	1602	*Hyrachyus*^***†***^	*eximius*^***†***^	metapodial	8	Bridger Basin	Bridger	Bridgerian
AMNH	1607	*Hyrachyus*^***†***^	*eximius*^***†***^	metapodial	1	Bridger	Bridger	Bridgerian
AMNH	1615	*Hyrachyus*^***†***^	*eximius*^***†***^	metapodial	1	Bridger Basin	Bridger	Bridgerian
AMNH	1621	*Hyrachyus*^***†***^	*eximius*^***†***^	metapodial	1	Bridger Basin	Bridger	Bridgerian
AMNH	1629	*Hyrachyus*^***†***^	*eximius*^***†***^	metapodial	5	Bridger Basin	Bridger	Bridgerian
AMNH	1645	*Hyrachyus*^***†***^	*eximius*^***†***^	metapodial	3	Twin Buttes	Bridger	Bridgerian
AMNH	5181	*Hyrachyus*^***†***^	*eximius*^***†***^	metapodial	3	Bridger Basin	Bridger	Bridgerian
AMNH	11693	*Hyrachyus*^***†***^	*eximius*^***†***^	metapodial	8	Bridger	Bridger	Bridgerian
AMNH	12353	*Hyrachyus*^***†***^	*eximius*^***†***^	metapodial	3	Cat-tail Spring	Bridger	Bridgerian
AMNH	12368	*Hyrachyus*^***†***^	*eximius*^***†***^	metapodial	3	Henry's Fork LT	Bridger	Bridgerian
AMNH	12665	*Hyrachyus*^***†***^	*eximius*^***†***^	metapodial	3	Grizzly Buttes	Bridger	Bridgerian
AMNH	12673	*Hyrachyus*^***†***^	*eximius*^***†***^	metapodial	5	Henry's Fork LT	Bridger	Bridgerian
AMNH	12674	*Hyrachyus*^***†***^	*eximius*^***†***^	metapodial	2	Bridger	Bridger	Bridgerian
AMNH	12675	*Hyrachyus*^***†***^	*eximius*^***†***^	metapodial	5	Kinney Ranch	Bridger	Bridgerian
AMNH	12765	*Hyrachyus*^***†***^	*eximius*^***†***^	metapodial	3	Black's Fork above Millersville	Bridger	Bridgerian
AMNH	93050	*Hyrachyus*^***†***^	*eximius*^***†***^	metapodial	1	Henry's Fork LT	Bridger	Bridgerian
AMNH	93060	*Hyrachyus*^***†***^	*eximius*^***†***^	metapodial	1	Bridger	Bridger	Bridgerian
AMNH	93061	*Hyrachyus*^***†***^	*eximius*^***†***^	metapodial	4	Grizzly Buttes East	Bridger	Bridgerian
AMNH	93064	*Hyrachyus*^***†***^	*eximius*^***†***^	metapodial	1	Henry's Fork LT	Bridger	Bridgerian
AMNH	105435	*Hyrachyus*^***†***^	*eximius*^***†***^	metapodial	1	Tabernacle Butte	Bridger	Bridgerian
AMNH	1644-A	*Hyrachyus*^***†***^	*eximius*^***†***^	metapodial	2	Bridger Basin	Bridger	Bridgerian
AMNH	1602	*Hyrachyus*^***†***^	*eximius*^***†***^	phalanx	3	Bridger Basin	Bridger	Bridgerian
AMNH	1626	*Hyrachyus*^***†***^	*eximius*^***†***^	phalanx	1	Bridger Basin	Bridger	Bridgerian
AMNH	1635	*Hyrachyus*^***†***^	*eximius*^***†***^	phalanx	1	Bridger Basin	Bridger	Bridgerian
AMNH	11693	*Hyrachyus*^***†***^	*eximius*^***†***^	phalanx	11	Bridger	Bridger	Bridgerian
AMNH	12353	*Hyrachyus*^***†***^	*eximius*^***†***^	phalanx	4	Cat-tail Spring	Bridger	Bridgerian
AMNH	12673	*Hyrachyus*^***†***^	*eximius*^***†***^	phalanx	6	Henry's Fork LT	Bridger	Bridgerian
AMNH	12675	*Hyrachyus*^***†***^	*eximius*^***†***^	phalanx	1	Black's Fork above Millersville	Bridger	Bridgerian
AMNH	93064	*Hyrachyus*^***†***^	*eximius*^***†***^	phalanx	4	Henry's Fork LT	Bridger	Bridgerian
AMNH	1644-A	*Hyrachyus*^***†***^	*eximius*^***†***^	phalanx	4	Bridger Basin	Bridger	Bridgerian
AMNH	1602	*Hyrachyus*^***†***^	*eximius*^***†***^	astragalus	1	Bridger Basin	Bridger	Bridgerian
AMNH	1607	*Hyrachyus*^***†***^	*eximius*^***†***^	astragalus	1	Bridger	Bridger	Bridgerian
AMNH	1607	*Hyrachyus*^***†***^	*eximius*^***†***^	podial	1	Bridger	Bridger	Bridgerian
AMNH	1615	*Hyrachyus*^***†***^	*eximius*^***†***^	astragalus	2	Bridger Basin	Bridger	Bridgerian
AMNH	1615	*Hyrachyus*^***†***^	*eximius*^***†***^	calcaneum	1	Bridger Basin	Bridger	Bridgerian
AMNH	1615	*Hyrachyus*^***†***^	*eximius*^***†***^	podial	2	Bridger Basin	Bridger	Bridgerian
AMNH	1621	*Hyrachyus*^***†***^	*eximius*^***†***^	calcaneum	1	Bridger Basin	Bridger	Bridgerian
AMNH	1626	*Hyrachyus*^***†***^	*eximius*^***†***^	podial	2	Bridger Basin	Bridger	Bridgerian
AMNH	1629	*Hyrachyus*^***†***^	*eximius*^***†***^	podial	1	Bridger Basin	Bridger	Bridgerian
AMNH	1635	*Hyrachyus*^***†***^	*eximius*^***†***^	astragalus	2	Bridger Basin	Bridger	Bridgerian
AMNH	1644	*Hyrachyus*^***†***^	*eximius*^***†***^	calcaneum	2	Bridger Basin	Bridger	Bridgerian
AMNH	1644	*Hyrachyus*^***†***^	*eximius*^***†***^	podial	2	Bridger Basin	Bridger	Bridgerian
AMNH	1645	*Hyrachyus*^***†***^	*eximius*^***†***^	astragalus	1	Twin Buttes	Bridger	Bridgerian
AMNH	1645	*Hyrachyus*^***†***^	*eximius*^***†***^	calcaneum	1	Twin Buttes	Bridger	Bridgerian
AMNH	1645	*Hyrachyus*^***†***^	*eximius*^***†***^	podial	2	Twin Buttes	Bridger	Bridgerian
AMNH	5056	*Hyrachyus*^***†***^	*eximius*^***†***^	astragalus	1	Grizzly Buttes	Bridger	Bridgerian
AMNH	5056	*Hyrachyus*^***†***^	*eximius*^***†***^	calcaneum	1	Grizzly Buttes	Bridger	Bridgerian
AMNH	5056	*Hyrachyus*^***†***^	*eximius*^***†***^	navicular	1	Grizzly Buttes	Bridger	Bridgerian
AMNH	5056	*Hyrachyus*^***†***^	*eximius*^***†***^	podial	3	Grizzly Buttes	Bridger	Bridgerian
AMNH	5181	*Hyrachyus*^***†***^	*eximius*^***†***^	magnum	1	Bridger Basin	Bridger	Bridgerian
AMNH	5196	*Hyrachyus*^***†***^	*eximius*^***†***^	astragalus	1	Bridger Basin	Bridger	Bridgerian
AMNH	5196	*Hyrachyus*^***†***^	*eximius*^***†***^	calcaneum	1	Bridger Basin	Bridger	Bridgerian
AMNH	11693	*Hyrachyus*^***†***^	*eximius*^***†***^	podial	9	Bridger	Bridger	Bridgerian
AMNH	11712	*Hyrachyus*^***†***^	*eximius*^***†***^	astragalus	2	Church Buttes	Bridger	Bridgerian
AMNH	11712	*Hyrachyus*^***†***^	*eximius*^***†***^	calcaneum	1	Church Buttes	Bridger	Bridgerian
AMNH	12179	*Hyrachyus*^***†***^	*eximius*^***†***^	astragalus	1	Mid. Cottonwood Cr.	Bridger	Bridgerian
AMNH	12179	*Hyrachyus*^***†***^	*eximius*^***†***^	calcaneum	1	Mid. Cottonwood Cr.	Bridger	Bridgerian
AMNH	12225	*Hyrachyus*^***†***^	*eximius*^***†***^	podial	1	Summer's Dry Cr'k	Bridger	Bridgerian
AMNH	12353	*Hyrachyus*^***†***^	*eximius*^***†***^	calcaneum	1	Cat-tail Spring	Bridger	Bridgerian
AMNH	12353	*Hyrachyus*^***†***^	*eximius*^***†***^	podial	6	Cat-tail Spring	Bridger	Bridgerian
AMNH	12353	*Hyrachyus*^***†***^	*eximius*^***†***^	sesimoid	3	Cat-tail Spring	Bridger	Bridgerian
AMNH	12368	*Hyrachyus*^***†***^	*eximius*^***†***^	podial	11	Henry's Fork LT	Bridger	Bridgerian
AMNH	12665	*Hyrachyus*^***†***^	*eximius*^***†***^	astragalus	2	Grizzly Buttes	Bridger	Bridgerian
AMNH	12665	*Hyrachyus*^***†***^	*eximius*^***†***^	calcaneum	1	Grizzly Buttes	Bridger	Bridgerian
AMNH	12665	*Hyrachyus*^***†***^	*eximius*^***†***^	podial	6	Grizzly Buttes	Bridger	Bridgerian
AMNH	12673	*Hyrachyus*^***†***^	*eximius*^***†***^	calcaneum	1	Henry's Fork LT	Bridger	Bridgerian
AMNH	12673	*Hyrachyus*^***†***^	*eximius*^***†***^	podial	10	Henry's Fork LT	Bridger	Bridgerian
AMNH	12674	*Hyrachyus*^***†***^	*eximius*^***†***^	astragalus	1	Bridger	Bridger	Bridgerian
AMNH	12674	*Hyrachyus*^***†***^	*eximius*^***†***^	calcaneum	1	Bridger	Bridger	Bridgerian
AMNH	12674	*Hyrachyus*^***†***^	*eximius*^***†***^	podial	6	Bridger	Bridger	Bridgerian
AMNH	12675	*Hyrachyus*^***†***^	*eximius*^***†***^	calcaneum	1	Kinney Ranch	Bridger	Bridgerian
AMNH	93050	*Hyrachyus*^***†***^	*eximius*^***†***^	podial	1	Henry's Fork LT	Bridger	Bridgerian
AMNH	93061	*Hyrachyus*^***†***^	*eximius*^***†***^	calcaneum	1	Grizzly Buttes East	Bridger	Bridgerian
AMNH	93061	*Hyrachyus*^***†***^	*eximius*^***†***^	calcaneum partial	1	Grizzly Buttes East	Bridger	Bridgerian
AMNH	93061	*Hyrachyus*^***†***^	*eximius*^***†***^	cuboid	1	Grizzly Buttes East	Bridger	Bridgerian
AMNH	93064	*Hyrachyus*^***†***^	*eximius*^***†***^	astragalus, calcaneum, and podial (articulated)	1	Henry's Fork LT	Bridger	Bridgerian
AMNH	93064	*Hyrachyus*^***†***^	*eximius*^***†***^	podial	1	Henry's Fork LT	Bridger	Bridgerian
AMNH	98726	*Hyrachyus*^***†***^	*eximius*^***†***^	pisiform	1	S. Hyopsodus Hill, Tabernacle Butte	Bridger	Bridgerian
AMNH	105435	*Hyrachyus*^***†***^	*eximius*^***†***^	astragalus	1	Tabernacle Butte	Bridger	Bridgerian
AMNH	12665-A	*Hyrachyus*^***†***^	*eximius*^***†***^	astragalus	1	Grizzly Buttes	Bridger	Bridgerian
AMNH	12665-A	*Hyrachyus*^***†***^	*eximius*^***†***^	calcaneum	1	Grizzly Buttes	Bridger	Bridgerian
AMNH	1536-A	*Hyrachyus*^***†***^	*eximius*^***†***^	astragalus	1	Bridger Basin	Bridger	Bridgerian
AMNH	1592-A	*Hyrachyus*^***†***^	*eximius*^***†***^	astragalus	1	Bridger Basin	Bridger	Bridgerian
AMNH	1596-A	*Hyrachyus*^***†***^	*eximius*^***†***^	astragalus	1	Bridger Basin	Bridger	Bridgerian
AMNH	1644-A	*Hyrachyus*^***†***^	*eximius*^***†***^	calcaneum	1	Bridger Basin	Bridger	Bridgerian
AMNH	1644-A	*Hyrachyus*^***†***^	*eximius*^***†***^	podial	4	Bridger Basin	Bridger	Bridgerian
AMNH	5065-B	*Hyrachyus*^***†***^	*eximius*^***†***^	calcaneum	3	Bridger Basin	Bridger	Bridgerian
AMNH	1602	*Hyrachyus*^***†***^	*eximius*^***†***^	axis	1	Bridger Basin	Bridger	Bridgerian
UCMP	32011	*Trigonias*^***†***^	*osborni*^***†***^	left femur	1	Figgins Quarry	White River	Chadronian
UCMP	32011	*Trigonias*^***†***^	*osborni*^***†***^	humerus	1	Figgins Quarry	White River	Chadronian
UCMP	32011	*Trigonias*^***†***^	*osborni*^***†***^	left humerus	2	Figgins Quarry	White River	Chadronian
UCMP	32011	*Trigonias*^***†***^	*osborni*^***†***^	left radius	1	Figgins Quarry	White River	Chadronian
UCMP	32012	*Trigonias*^***†***^	*osborni*^***†***^	left ulna	1	Figgins Quarry	White River	Chadronian
UCMP	32011	*Trigonias*^***†***^	*osborni*^***†***^	left scapula	2	Figgins Quarry	White River	Chadronian
UCMP	32011	*Trigonias*^***†***^	*osborni*^***†***^	left tibia	2	Figgins Quarry	White River	Chadronian
UCMP	32011	*Trigonias*^***†***^	*osborni*^***†***^	left fibula	1	Figgins Quarry	White River	Chadronian
UCMP	32012	*Trigonias*^***†***^	*osborni*^***†***^	left tibia	1	Figgins Quarry	White River	Chadronian
UCMP	32011	*Trigonias*^***†***^	*osborni*^***†***^	patella	1	Figgins Quarry	White River	Chadronian
UCMP	32011	*Trigonias*^***†***^	*osborni*^***†***^	right tibiofibula	1	Figgins Quarry	White River	Chadronian
UCMP	32011	*Trigonias*^***†***^	*osborni*^***†***^	right ulna	1	Figgins Quarry	White River	Chadronian
UCMP	32012	*Trigonias*^***†***^	*osborni*^***†***^	right radius	1	Figgins Quarry	White River	Chadronian
UCMP	32011	*Trigonias*^***†***^	*osborni*^***†***^	right ulna	1	Figgins Quarry	White River	Chadronian
UCMP	32011	*Trigonias*^***†***^	*osborni*^***†***^	left femur	1	Figgins Quarry	White River	Chadronian
UCMP	32011	*Trigonias*^***†***^	*osborni*^***†***^	patellae	1	Figgins Quarry	White River	Chadronian
UCMP	32011	*Trigonias*^***†***^	*osborni*^***†***^	left humerus	1	Figgins Quarry	White River	Chadronian
UCMP	32011	*Trigonias*^***†***^	*osborni*^***†***^	right humerus	2	Figgins Quarry	White River	Chadronian
UCMP	32011	*Trigonias*^***†***^	*osborni*^***†***^	metapodial	23	Figgins Quarry	White River	Chadronian
UCMP	32011	*Trigonias*^***†***^	*osborni*^***†***^	metatarsal	1	Figgins Quarry	White River	Chadronian
UCMP	32011	*Trigonias*^***†***^	*osborni*^***†***^	left metatarsal	2	Figgins Quarry	White River	Chadronian
UCMP	32011	*Trigonias*^***†***^	*osborni*^***†***^	metatarsal	4	Figgins Quarry	White River	Chadronian
UCMP	32011	*Trigonias*^***†***^	*osborni*^***†***^	metapodial	1	Figgins Quarry	White River	Chadronian
UCMP	32011	*Trigonias*^***†***^	*osborni*^***†***^	phalanx	33	Figgins Quarry	White River	Chadronian
UCMP	32011	*Trigonias*^***†***^	*osborni*^***†***^	phalanx 3	1	Figgins Quarry	White River	Chadronian
UCMP	32011	*Trigonias*^***†***^	*osborni*^***†***^	astragalus	1	Figgins Quarry	White River	Chadronian
UCMP	32011	*Trigonias*^***†***^	*osborni*^***†***^	calcaneum	2	Figgins Quarry	White River	Chadronian
UCMP	32011	*Trigonias*^***†***^	*osborni*^***†***^	cuneiform	8	Figgins Quarry	White River	Chadronian
UCMP	32011	*Trigonias*^***†***^	*osborni*^***†***^	left podial	1	Figgins Quarry	White River	Chadronian
UCMP	32011	*Trigonias*^***†***^	*osborni*^***†***^	carpal	1	Figgins Quarry	White River	Chadronian
UCMP	32011	*Trigonias*^***†***^	*osborni*^***†***^	podial	1	Figgins Quarry	White River	Chadronian
UCMP	32011	*Trigonias*^***†***^	*osborni*^***†***^	pisiform	4	Figgins Quarry	White River	Chadronian
UCMP	32011	*Trigonias*^***†***^	*osborni*^***†***^	podial	2	Figgins Quarry	White River	Chadronian
UCMP	32011	*Trigonias*^***†***^	*osborni*^***†***^	navicular	3	Figgins Quarry	White River	Chadronian
UCMP	32011	*Trigonias*^***†***^	*osborni*^***†***^	left pisiform	1	Figgins Quarry	White River	Chadronian
UCMP	32011	*Trigonias*^***†***^	*osborni*^***†***^	right pisiform	1	Figgins Quarry	White River	Chadronian
AMNH	144571	*Menoceras*^***†***^	*arikarense*^***†***^	scapula	1	Agate Spring Quarry	Harrison	Arikareean
AMNH	144572	*Menoceras*^***†***^	*arikarense*^***†***^	scapula	1	Agate Spring Quarry	Harrison	Arikareean
AMNH	144573	*Menoceras*^***†***^	*arikarense*^***†***^	scapula	1	Agate Spring Quarry	Harrison	Arikareean
AMNH	144574	*Menoceras*^***†***^	*arikarense*^***†***^	scapula	1	Agate Spring Quarry	Harrison	Arikareean
AMNH	144575	*Menoceras*^***†***^	*arikarense*^***†***^	scapula	1	Agate Spring Quarry	Harrison	Arikareean
AMNH	144576	*Menoceras*^***†***^	*arikarense*^***†***^	scapula	1	Agate Spring Quarry	Harrison	Arikareean
AMNH	144577	*Menoceras*^***†***^	*arikarense*^***†***^	scapula	1	Agate Spring Quarry	Harrison	Arikareean
AMNH	144578	*Menoceras*^***†***^	*arikarense*^***†***^	scapula	1	Agate Spring Quarry	Harrison	Arikareean
AMNH	144579	*Menoceras*^***†***^	*arikarense*^***†***^	scapula	1	Agate Spring Quarry	Harrison	Arikareean
AMNH	14213	*Menoceras*^***†***^	*arikarense*^***†***^	humerus	1	Agate Spring Quarry	Harrison	Arikareean
AMNH	14213	*Menoceras*^***†***^	*arikarense*^***†***^	radius-ulna	1	Agate Spring Quarry	Harrison	Arikareean
AMNH	14214	*Menoceras*^***†***^	*arikarense*^***†***^	humerus	1	Agate Spring Quarry	Harrison	Arikareean
AMNH	14214	*Menoceras*^***†***^	*arikarense*^***†***^	proximal tibia	1	Agate Spring Quarry	Harrison	Arikareean
AMNH	22486	*Menoceras*^***†***^	*arikarense*^***†***^	femur	1	Agate Spring Quarry	Harrison	Arikareean
AMNH	22486	*Menoceras*^***†***^	*arikarense*^***†***^	humerus	1	Agate Spring Quarry	Harrison	Arikareean
AMNH	22486	*Menoceras*^***†***^	*arikarense*^***†***^	radius	1	Agate Spring Quarry	Harrison	Arikareean
AMNH	22487	*Menoceras*^***†***^	*arikarense*^***†***^	fibula	1	Agate Spring Quarry	Harrison	Arikareean
AMNH	22487	*Menoceras*^***†***^	*arikarense*^***†***^	humerus	1	Agate Spring Quarry	Harrison	Arikareean
AMNH	22487	*Menoceras*^***†***^	*arikarense*^***†***^	ulna	1	Agate Spring Quarry	Harrison	Arikareean
AMNH	144597	*Menoceras*^***†***^	*arikarense*^***†***^	ulna	1	Agate Spring Quarry	Harrison	Arikareean
AMNH	144598	*Menoceras*^***†***^	*arikarense*^***†***^	ulna	1	Agate Spring Quarry	Harrison	Arikareean
AMNH	144599	*Menoceras*^***†***^	*arikarense*^***†***^	ulna	1	Agate Spring Quarry	Harrison	Arikareean
AMNH	144600	*Menoceras*^***†***^	*arikarense*^***†***^	ulna	1	Agate Spring Quarry	Harrison	Arikareean
AMNH	144602	*Menoceras*^***†***^	*arikarense*^***†***^	ulna	1	Agate Spring Quarry	Harrison	Arikareean
AMNH	144603	*Menoceras*^***†***^	*arikarense*^***†***^	ulna	1	Agate Spring Quarry	Harrison	Arikareean
AMNH	144604	*Menoceras*^***†***^	*arikarense*^***†***^	ulna	1	Agate Spring Quarry	Harrison	Arikareean
AMNH	144605	*Menoceras*^***†***^	*arikarense*^***†***^	ulna	1	Agate Spring Quarry	Harrison	Arikareean
AMNH	144606	*Menoceras*^***†***^	*arikarense*^***†***^	ulna	1	Agate Spring Quarry	Harrison	Arikareean
AMNH	144607	*Menoceras*^***†***^	*arikarense*^***†***^	ulna	1	Agate Spring Quarry	Harrison	Arikareean
AMNH	144608	*Menoceras*^***†***^	*arikarense*^***†***^	ulna	1	Agate Spring Quarry	Harrison	Arikareean
AMNH	144580	*Menoceras*^***†***^	*arikarense*^***†***^	humerus	1	Agate Spring Quarry	Harrison	Arikareean
AMNH	144581	*Menoceras*^***†***^	*arikarense*^***†***^	humerus	1	Agate Spring Quarry	Harrison	Arikareean
AMNH	144582	*Menoceras*^***†***^	*arikarense*^***†***^	humerus	1	Agate Spring Quarry	Harrison	Arikareean
AMNH	144583	*Menoceras*^***†***^	*arikarense*^***†***^	humerus	1	Agate Spring Quarry	Harrison	Arikareean
AMNH	144585	*Menoceras*^***†***^	*arikarense*^***†***^	humerus	1	Agate Spring Quarry	Harrison	Arikareean
AMNH	144586	*Menoceras*^***†***^	*arikarense*^***†***^	humerus	1	Agate Spring Quarry	Harrison	Arikareean
AMNH	144587	*Menoceras*^***†***^	*arikarense*^***†***^	humerus	1	Agate Spring Quarry	Harrison	Arikareean
AMNH	144588	*Menoceras*^***†***^	*arikarense*^***†***^	humerus	1	Agate Spring Quarry	Harrison	Arikareean
AMNH	144589	*Menoceras*^***†***^	*arikarense*^***†***^	humerus	1	Agate Spring Quarry	Harrison	Arikareean
AMNH	144590	*Menoceras*^***†***^	*arikarense*^***†***^	humerus	1	Agate Spring Quarry	Harrison	Arikareean
AMNH	144591	*Menoceras*^***†***^	*arikarense*^***†***^	humerus	1	Agate Spring Quarry	Harrison	Arikareean
AMNH	144592	*Menoceras*^***†***^	*arikarense*^***†***^	humerus	1	Agate Spring Quarry	Harrison	Arikareean
AMNH	144593	*Menoceras*^***†***^	*arikarense*^***†***^	humerus	1	Agate Spring Quarry	Harrison	Arikareean
AMNH	144594	*Menoceras*^***†***^	*arikarense*^***†***^	humerus	1	Agate Spring Quarry	Harrison	Arikareean
AMNH	144595	*Menoceras*^***†***^	*arikarense*^***†***^	humerus	1	Agate Spring Quarry	Harrison	Arikareean
AMNH	144596	*Menoceras*^***†***^	*arikarense*^***†***^	humerus	1	Agate Spring Quarry	Harrison	Arikareean
AMNH	144609	*Menoceras*^***†***^	*arikarense*^***†***^	radius	1	Agate Spring Quarry	Harrison	Arikareean
AMNH	144610	*Menoceras*^***†***^	*arikarense*^***†***^	radius	1	Agate Spring Quarry	Harrison	Arikareean
AMNH	144611	*Menoceras*^***†***^	*arikarense*^***†***^	radius	1	Agate Spring Quarry	Harrison	Arikareean
AMNH	144612	*Menoceras*^***†***^	*arikarense*^***†***^	radius	1	Agate Spring Quarry	Harrison	Arikareean
AMNH	144616	*Menoceras*^***†***^	*arikarense*^***†***^	radius	1	Agate Spring Quarry	Harrison	Arikareean
AMNH	144613	*Menoceras*^***†***^	*arikarense*^***†***^	radius	1	Agate Spring Quarry	Harrison	Arikareean
AMNH	144614	*Menoceras*^***†***^	*arikarense*^***†***^	radius	1	Agate Spring Quarry	Harrison	Arikareean
AMNH	144615	*Menoceras*^***†***^	*arikarense*^***†***^	radius	1	Agate Spring Quarry	Harrison	Arikareean
AMNH	144617	*Menoceras*^***†***^	*arikarense*^***†***^	radius	1	Agate Spring Quarry	Harrison	Arikareean
AMNH	144618	*Menoceras*^***†***^	*arikarense*^***†***^	radius	1	Agate Spring Quarry	Harrison	Arikareean
AMNH	144619	*Menoceras*^***†***^	*arikarense*^***†***^	radius	1	Agate Spring Quarry	Harrison	Arikareean
AMNH	144622	*Menoceras*^***†***^	*arikarense*^***†***^	radius	1	Agate Spring Quarry	Harrison	Arikareean
AMNH	144621	*Menoceras*^***†***^	*arikarense*^***†***^	radius	1	Agate Spring Quarry	Harrison	Arikareean
AMNH	144620	*Menoceras*^***†***^	*arikarense*^***†***^	radius	1	Agate Spring Quarry	Harrison	Arikareean
AMNH	144623	*Menoceras*^***†***^	*arikarense*^***†***^	radius	1	Agate Spring Quarry	Harrison	Arikareean
AMNH	144625	*Menoceras*^***†***^	*arikarense*^***†***^	radius	1	Agate Spring Quarry	Harrison	Arikareean
AMNH	144624	*Menoceras*^***†***^	*arikarense*^***†***^	radius	1	Agate Spring Quarry	Harrison	Arikareean
AMNH	144633	*Menoceras*^***†***^	*arikarense*^***†***^	femur	1	Agate Spring Quarry	Harrison	Arikareean
AMNH	144634	*Menoceras*^***†***^	*arikarense*^***†***^	femur	1	Agate Spring Quarry	Harrison	Arikareean
AMNH	144635	*Menoceras*^***†***^	*arikarense*^***†***^	tibia	1	Agate Spring Quarry	Harrison	Arikareean
AMNH	144636	*Menoceras*^***†***^	*arikarense*^***†***^	fibula	1	Agate Spring Quarry	Harrison	Arikareean
AMNH	144584	*Menoceras*^***†***^	*arikarense*^***†***^	humerus	1	Agate Spring Quarry	Harrison	Arikareean
AMNH	86090	*Menoceras*^***†***^	*arikarense*^***†***^	partial metapodials	1	Agate Spring Quarry	Harrison	Arikareean
AMNH	144626	*Menoceras*^***†***^	*arikarense*^***†***^	metapodial	1	Agate Spring Quarry	Harrison	Arikareean
AMNH	144627	*Menoceras*^***†***^	*arikarense*^***†***^	metapodial	1	Agate Spring Quarry	Harrison	Arikareean
AMNH	144628	*Menoceras*^***†***^	*arikarense*^***†***^	metapodial	1	Agate Spring Quarry	Harrison	Arikareean
AMNH	86090	*Menoceras*^***†***^	*arikarense*^***†***^	left phalanx	1	Agate Spring Quarry	Harrison	Arikareean
AMNH	86091	*Menoceras*^***†***^	*arikarense*^***†***^	right phalanx	1	Agate Spring Quarry	Harrison	Arikareean
AMNH	86092	*Menoceras*^***†***^	*arikarense*^***†***^	phalanx	1	Agate Spring Quarry	Harrison	Arikareean
AMNH	144629	*Menoceras*^***†***^	*arikarense*^***†***^	phalanx	1	Agate Spring Quarry	Harrison	Arikareean
AMNH	144632	*Menoceras*^***†***^	*arikarense*^***†***^	phalanx	1	Agate Spring Quarry	Harrison	Arikareean
AMNH	144630	*Menoceras*^***†***^	*arikarense*^***†***^	phalanx	1	Agate Spring Quarry	Harrison	Arikareean
AMNH	144631	*Menoceras*^***†***^	*arikarense*^***†***^	phalanx	1	Agate Spring Quarry	Harrison	Arikareean
AMNH	86090	*Menoceras*^***†***^	*arikarense*^***†***^	astragalus	1	Agate Spring Quarry	Harrison	Arikareean
AMNH	86090	*Menoceras*^***†***^	*arikarense*^***†***^	calcaneum	1	Agate Spring Quarry	Harrison	Arikareean
AMNH	86090	*Menoceras*^***†***^	*arikarense*^***†***^	podial	2	Agate Spring Quarry	Harrison	Arikareean
UW	52334	*Diceratherium*^***†***^	*niobrarense*^***†***^	scapula	1	John Day Formation	John Day Formation	Whitneyan
UW	58203	*Diceratherium*^***†***^	*niobrarense*^***†***^	scapula	1	south canyon	John Day Formation	Whitneyan
UCMP	2289	*Diceratherium*^***†***^	*niobrarense*^***†***^	pelvis frag	1	Logan Butte	John Day Formation	Whitneyan
UW	26414	*Diceratherium*^***†***^	*niobrarense*^***†***^	patella	1	Blue Basin	John Day Formation	Whitneyan
UW	26536	*Diceratherium*^***†***^	*niobrarense*^***†***^	distal femur	1	John Day Formation	John Day Formation	Whitneyan
UW	28151	*Diceratherium*^***†***^	*niobrarense*^***†***^	femoral head	1	John Day Formation	John Day Formation	Whitneyan
UW	52334	*Diceratherium*^***†***^	*niobrarense*^***†***^	distal femur	1	John Day Formation	John Day Formation	Whitneyan
UW	53301	*Diceratherium*^***†***^	*niobrarense*^***†***^	tibia	1	picture gorge 14	John Day Formation	Whitneyan
UW	53302	*Diceratherium*^***†***^	*niobrarense*^***†***^	proximal tibia	1	picture gorge 51	John Day Formation	Whitneyan
UW	53323	*Diceratherium*^***†***^	*niobrarense*^***†***^	tibia (juvenile)	1	picture gorge 54	John Day Formation	Whitneyan
UW	53324	*Diceratherium*^***†***^	*niobrarense*^***†***^	femur (juvenile)	1	picture gorge 54	John Day Formation	Whitneyan
UW	53325	*Diceratherium*^***†***^	*niobrarense*^***†***^	distal femur	1	picture gorge 20	John Day Formation	Whitneyan
UW	53430	*Diceratherium*^***†***^	*niobrarense*^***†***^	distal femur	1	picture gorge 54	John Day Formation	Whitneyan
UW	55086	*Diceratherium*^***†***^	*niobrarense*^***†***^	tibia	1	North Wash Level 5	John Day Formation	Whitneyan
UW	58755	*Diceratherium*^***†***^	*niobrarense*^***†***^	tibia	1	Blue Canyon	John Day Formation	Whitneyan
UW	58755	*Diceratherium*^***†***^	*niobrarense*^***†***^	limb bone	2	Blue Canyon	John Day Formation	Whitneyan
UW	533315	*Diceratherium*^***†***^	*niobrarense*^***†***^	humerus	1	picture gorge 12	John Day Formation	Whitneyan
UCMP	145	*Diceratherium*^***†***^	*niobrarense*^***†***^	distal femur and podial	1	John Day Whitneyan General	John Day Formation	Whitneyan
UCMP	566	*Diceratherium*^***†***^	*niobrarense*^***†***^	proximal radioulna	1	John Day Whitneyan General	John Day Formation	Whitneyan
UCMP	75260	*Diceratherium*^***†***^	*niobrarense*^***†***^	proximal humerus	1	South Canyon 2	John Day Formation	Whitneyain
UCMP	75261	*Diceratherium*^***†***^	*niobrarense*^***†***^	distal humerus	1	South Canyon 2	John Day Formation	Whitneyain
UCMP	75261	*Diceratherium*^***†***^	*niobrarense*^***†***^	proximal humerus	1	South Canyon 2	John Day Formation	Whitneyain
UCMP	75282	*Diceratherium*^***†***^	*niobrarense*^***†***^	femur	1	South Canyon 2	John Day Formation	Whitneyain
UCMP	M1691	*Diceratherium*^***†***^	*niobrarense*^***†***^	distal femur	1	Logan Butte	John Day Formation	Whitneyain
UCMP	M1691	*Diceratherium*^***†***^	*niobrarense*^***†***^	partial fibula	1	Logan Butte	John Day Formation	Whitneyain
UCMP	M1691	*Diceratherium*^***†***^	*niobrarense*^***†***^	tibia	1	Logan Butte	John Day Formation	Whitneyain
UCMP	M2107	*Diceratherium*^***†***^	*niobrarense*^***†***^	distal femur	1	Seigfried's 4	John Day Formation	Whitneyain
UW	26563	*Diceratherium*^***†***^	*niobrarense*^***†***^	distal metapodial	1	John Day Formation	John Day Formation	Whitneyain
UW	26879	*Diceratherium*^***†***^	*niobrarense*^***†***^	distal metapodial	1	John Day Formation	John Day Formation	Whitneyain
UW	43529	*Diceratherium*^***†***^	*niobrarense*^***†***^	metatarsal	1	John Day Formation	John Day Formation	Whitneyain
UW	52334	*Diceratherium*^***†***^	*niobrarense*^***†***^	metapodial	1	picture gorge	John Day Formation	Whitneyain
UW	52334	*Diceratherium*^***†***^	*niobrarense*^***†***^	distal metapodial	1	John Day Formation	John Day Formation	Whitneyain
UW	53322	*Diceratherium*^***†***^	*niobrarense*^***†***^	metapodial	1	picture gorge 16	John Day Formation	Whitneyain
UW	55086	*Diceratherium*^***†***^	*niobrarense*^***†***^	metatarsal	1	North Wash Level 5	John Day Formation	Whitneyain
UCMP	145	*Diceratherium*^***†***^	*niobrarense*^***†***^	right metatarsal 3	1	John Day Whitneyan General	John Day Formation	Whitneyain
UCMP	M1691	*Diceratherium*^***†***^	*niobrarense*^***†***^	left metapodial	1	Logan Butte	John Day Formation	Whitneyain
UCMP	M1691	*Diceratherium*^***†***^	*niobrarense*^***†***^	metapodial	5	Logan Butte	John Day Formation	Whitneyain
UW	52334	*Diceratherium*^***†***^	*niobrarense*^***†***^	phalanx	2	John Day Formation	John Day Formation	Whitneyain
UW	53322	*Diceratherium*^***†***^	*niobrarense*^***†***^	phalanx	1	picture gorge 16	John Day Formation	Whitneyain
UCMP	788	*Diceratherium*^***†***^	*niobrarense*^***†***^	phalanx	1	John Day Whitneyan General	John Day Formation	Whitneyain
UCMP	75403	*Diceratherium*^***†***^	*niobrarense*^***†***^	medial phalanx	1	South Canyon 2	John Day Formation	Whitneyain
UCMP	M1691	*Diceratherium*^***†***^	*niobrarense*^***†***^	phalanx	3	Logan Butte	John Day Formation	Whitneyain
UW	52334	*Diceratherium*^***†***^	*niobrarense*^***†***^	podial	2	John Day Formation	John Day Formation	Whitneyain
UW	53322	*Diceratherium*^***†***^	*niobrarense*^***†***^	podial	2	picture gorge 16	John Day Formation	Whitneyain
UW	54947	*Diceratherium*^***†***^	*niobrarense*^***†***^	astragalus and partial calcanium	1	picture gorge 8 6' up	John Day Formation	Whitneyain
UW	54947	*Diceratherium*^***†***^	*niobrarense*^***†***^	podial	2	picture gorge 8 6' up	John Day Formation	Whitneyain
UW	55086	*Diceratherium*^***†***^	*niobrarense*^***†***^	podial	2	North Wash Level 5	John Day Formation	Whitneyain
UW	75665	*Diceratherium*^***†***^	*niobrarense*^***†***^	distal podial	1	picture gorge 29	John Day Formation	Whitneyain
UCMP	788	*Diceratherium*^***†***^	*niobrarense*^***†***^	tarsal	1	John Day Whitneyan General	John Day Formation	Whitneyain
UCMP	75033	*Diceratherium*^***†***^	*niobrarense*^***†***^	navicular	1	South Canyon 2	John Day Formation	Whitneyain
UCMP	75035	*Diceratherium*^***†***^	*niobrarense*^***†***^	lunar	1	South Canyon 2	John Day Formation	Whitneyain
UCMP	75120	*Diceratherium*^***†***^	*niobrarense*^***†***^	astragalus	1	South Canyon 2	John Day Formation	Whitneyain
UCMP	76104	*Diceratherium*^***†***^	*niobrarense*^***†***^	podial	2	South Canyon 2	John Day Formation	Whitneyain
UCMP	75260	*Diceratherium*^***†***^	*niobrarense*^***†***^	podial	1	South Canyon 2	John Day Formation	Whitneyain
UCMP	75348	*Diceratherium*^***†***^	*niobrarense*^***†***^	middle podial	1	South Canyon 2	John Day Formation	Whitneyain
UCMP	76104	*Diceratherium*^***†***^	*niobrarense*^***†***^	right podial	1	South Canyon 2	John Day Formation	Whitneyain
UCMP	M1691	*Diceratherium*^***†***^	*niobrarense*^***†***^	articulated astragalus and calcaneum	1	Logan Butte	John Day Formation	Whitneyain
UW	58755	*Diceratherium*^***†***^	*niobrarense*^***†***^	centrum	1	Blue Canyon	John Day Formation	Whitneyain
UCMP	22552	*Aphelops*^***†***^	*mutilis*^***†***^	left femur	1	Coffee Ranch Quarry 2	Ogalla Group	Hemphilian
UCMP	30166	*Aphelops*^***†***^	*mutilis*^***†***^	tibia	1	Higgins Quarry A	Ogalla Group	Hemphilian
UCMP	30266	*Aphelops*^***†***^	*mutilis*^***†***^	dist end of ulna	1	Coffee Ranch Quarry 2	Ogalla Group	Hemphilian
UCMP	30266	*Aphelops*^***†***^	*mutilis*^***†***^	distal humerus	1	Coffee Ranch Quarry 2	Ogalla Group	Hemphilian
UCMP	30266	*Aphelops*^***†***^	*mutilis*^***†***^	left femur	1	Coffee Ranch Quarry 2	Ogalla Group	Hemphilian
UCMP	30266	*Aphelops*^***†***^	*mutilis*^***†***^	partial distal tibia	1	Coffee Ranch Quarry 2	Ogalla Group	Hemphilian
UCMP	30266	*Aphelops*^***†***^	*mutilis*^***†***^	patella	1	Coffee Ranch Quarry 2	Ogalla Group	Hemphilian
UCMP	30266	*Aphelops*^***†***^	*mutilis*^***†***^	tibia	2	Coffee Ranch Quarry 2	Ogalla Group	Hemphilian
UCMP	30266	*Aphelops*^***†***^	*mutilis*^***†***^	ulna	2	Coffee Ranch Quarry 2	Ogalla Group	Hemphilian
UCMP	30267	*Aphelops*^***†***^	*mutilis*^***†***^	partial distal tibia	1	Coffee Ranch Quarry 2	Ogalla Group	Hemphilian
UCMP	30267	*Aphelops*^***†***^	*mutilis*^***†***^	patella	1	Coffee Ranch Quarry 2	Ogalla Group	Hemphilian
UCMP	30268	*Aphelops*^***†***^	*mutilis*^***†***^	patella	1	Coffee Ranch Quarry 2	Ogalla Group	Hemphilian
UCMP	30612	*Aphelops*^***†***^	*mutilis*^***†***^	femur	1	Higgins Quarry A	Ogalla Group	Hemphilian
UCMP	30613	*Aphelops*^***†***^	*mutilis*^***†***^	humerus	1	Higgins Quarry A	Ogalla Group	Hemphilian
UCMP	31117	*Aphelops*^***†***^	*mutilis*^***†***^	ulna	1	Coffee Ranch Quarry 2	Ogalla Group	Hemphilian
UCMP	31118	*Aphelops*^***†***^	*mutilis*^***†***^	tibia	1	Coffee Ranch Quarry 2	Ogalla Group	Hemphilian
UCMP	31119	*Aphelops*^***†***^	*mutilis*^***†***^	humerus	1	Coffee Ranch Quarry 2	Ogalla Group	Hemphilian
UCMP	31120	*Aphelops*^***†***^	*mutilis*^***†***^	humerus	1	Coffee Ranch Quarry 2	Ogalla Group	Hemphilian
UCMP	31121	*Aphelops*^***†***^	*mutilis*^***†***^	humerus	1	Coffee Ranch Quarry 2	Ogalla Group	Hemphilian
UCMP	31122	*Aphelops*^***†***^	*mutilis*^***†***^	humerus	1	Coffee Ranch Quarry 2	Ogalla Group	Hemphilian
UCMP	31127	*Aphelops*^***†***^	*mutilis*^***†***^	patella	1	Coffee Ranch Quarry 2	Ogalla Group	Hemphilian
UCMP	31128	*Aphelops*^***†***^	*mutilis*^***†***^	patella	1	Coffee Ranch Quarry 2	Ogalla Group	Hemphilian
UCMP	31129	*Aphelops*^***†***^	*mutilis*^***†***^	patella	1	Coffee Ranch Quarry 2	Ogalla Group	Hemphilian
UCMP	30266	*Aphelops*^***†***^	*mutilis*^***†***^	partial metacarpal	4	Coffee Ranch Quarry 2	Ogalla Group	Hemphilian
UCMP	30266	*Aphelops*^***†***^	*mutilis*^***†***^	metacarpal	5	Coffee Ranch Quarry 2	Ogalla Group	Hemphilian
UCMP	31123	*Aphelops*^***†***^	*mutilis*^***†***^	metacarpal	3	Coffee Ranch Quarry 2	Ogalla Group	Hemphilian
UCMP	32066	*Aphelops*^***†***^	*mutilis*^***†***^	metatarsal	11	Coffee Ranch Quarry 2	Ogalla Group	Hemphilian
UCMP	32067	*Aphelops*^***†***^	*mutilis*^***†***^	metatarsal	3	Coffee Ranch Quarry 2	Ogalla Group	Hemphilian
UCMP	32068	*Aphelops*^***†***^	*mutilis*^***†***^	metatarsal	3	Coffee Ranch Quarry 2	Ogalla Group	Hemphilian
UCMP	30266	*Aphelops*^***†***^	*mutilis*^***†***^	phalanx 3	1	Coffee Ranch Quarry 2	Ogalla Group	Hemphilian
UCMP	30267	*Aphelops*^***†***^	*mutilis*^***†***^	phalanx	1	Coffee Ranch Quarry 2	Ogalla Group	Hemphilian
UCMP	30268	*Aphelops*^***†***^	*mutilis*^***†***^	phalanx	1	Coffee Ranch Quarry 2	Ogalla Group	Hemphilian
UCMP	30269	*Aphelops*^***†***^	*mutilis*^***†***^	phalanx	1	Coffee Ranch Quarry 2	Ogalla Group	Hemphilian
UCMP	30270	*Aphelops*^***†***^	*mutilis*^***†***^	phalanx	1	Coffee Ranch Quarry 2	Ogalla Group	Hemphilian
UCMP	30271	*Aphelops*^***†***^	*mutilis*^***†***^	phalanx	1	Coffee Ranch Quarry 2	Ogalla Group	Hemphilian
UCMP	30272	*Aphelops*^***†***^	*mutilis*^***†***^	phalanx	1	Coffee Ranch Quarry 2	Ogalla Group	Hemphilian
UCMP	30273	*Aphelops*^***†***^	*mutilis*^***†***^	phalanx	1	Coffee Ranch Quarry 2	Ogalla Group	Hemphilian
UCMP	30274	*Aphelops*^***†***^	*mutilis*^***†***^	phalanx	1	Coffee Ranch Quarry 2	Ogalla Group	Hemphilian
UCMP	30275	*Aphelops*^***†***^	*mutilis*^***†***^	phalanx	1	Coffee Ranch Quarry 2	Ogalla Group	Hemphilian
UCMP	31127	*Aphelops*^***†***^	*mutilis*^***†***^	phalanx	1	Coffee Ranch Quarry 2	Ogalla Group	Hemphilian
UCMP	31128	*Aphelops*^***†***^	*mutilis*^***†***^	phalanx	1	Coffee Ranch Quarry 2	Ogalla Group	Hemphilian
UCMP	31129	*Aphelops*^***†***^	*mutilis*^***†***^	phalanx	1	Coffee Ranch Quarry 2	Ogalla Group	Hemphilian
UCMP	31130	*Aphelops*^***†***^	*mutilis*^***†***^	phalanx	1	Coffee Ranch Quarry 2	Ogalla Group	Hemphilian
UCMP	31131	*Aphelops*^***†***^	*mutilis*^***†***^	phalanx	1	Coffee Ranch Quarry 2	Ogalla Group	Hemphilian
UCMP	31132	*Aphelops*^***†***^	*mutilis*^***†***^	phalanx	1	Coffee Ranch Quarry 2	Ogalla Group	Hemphilian
UCMP	31133	*Aphelops*^***†***^	*mutilis*^***†***^	phalanx	1	Coffee Ranch Quarry 2	Ogalla Group	Hemphilian
UCMP	30266	*Aphelops*^***†***^	*mutilis*^***†***^	carpals	6	Coffee Ranch Quarry 2	Ogalla Group	Hemphilian
UCMP	30266	*Aphelops*^***†***^	*mutilis*^***†***^	right calcaneum	1	Coffee Ranch Quarry 2	Ogalla Group	Hemphilian
UCMP	30266	*Aphelops*^***†***^	*mutilis*^***†***^	carpal	1	Coffee Ranch Quarry 2	Ogalla Group	Hemphilian
UCMP	30266	*Aphelops*^***†***^	*mutilis*^***†***^	tarsal	1	Coffee Ranch Quarry 2	Ogalla Group	Hemphilian
UCMP	30267	*Aphelops*^***†***^	*mutilis*^***†***^	calcaneum	1	Coffee Ranch Quarry 3	Ogalla Group	Hemphilian
UCMP	30267	*Aphelops*^***†***^	*mutilis*^***†***^	tarsal	1	Coffee Ranch Quarry 4	Ogalla Group	Hemphilian
UCMP	30268	*Aphelops*^***†***^	*mutilis*^***†***^	tarsal	1	Coffee Ranch Quarry 5	Ogalla Group	Hemphilian
UCMP	30269	*Aphelops*^***†***^	*mutilis*^***†***^	tarsal	1	Coffee Ranch Quarry 6	Ogalla Group	Hemphilian
UCMP	30270	*Aphelops*^***†***^	*mutilis*^***†***^	tarsal	1	Coffee Ranch Quarry 7	Ogalla Group	Hemphilian
UCMP	31124	*Aphelops*^***†***^	*mutilis*^***†***^	astragalus	1	Coffee Ranch Quarry 2	Ogalla Group	Hemphilian
UCMP	31125	*Aphelops*^***†***^	*mutilis*^***†***^	astragalus	1	Coffee Ranch Quarry 2	Ogalla Group	Hemphilian
UCMP	31125	*Aphelops*^***†***^	*mutilis*^***†***^	calcaneum	1	Coffee Ranch Quarry 2	Ogalla Group	Hemphilian
UCMP	31125	*Aphelops*^***†***^	*mutilis*^***†***^	calcaneum	1	Coffee Ranch Quarry 2	Ogalla Group	Hemphilian
UCMP	31126	*Aphelops*^***†***^	*mutilis*^***†***^	calcaneum	1	Coffee Ranch Quarry 2	Ogalla Group	Hemphilian
UCMP	31127	*Aphelops*^***†***^	*mutilis*^***†***^	calcaneum	1	Coffee Ranch Quarry 2	Ogalla Group	Hemphilian
UCMP	31127	*Aphelops*^***†***^	*mutilis*^***†***^	carpal	9	Coffee Ranch Quarry 2	Ogalla Group	Hemphilian
UCMP	31128	*Aphelops*^***†***^	*mutilis*^***†***^	tarsal	1	Coffee Ranch Quarry 2	Ogalla Group	Hemphilian
UCMP	32066	*Aphelops*^***†***^	*mutilis*^***†***^	podial	1	Coffee Ranch Quarry 2	Ogalla Group	Hemphilian
UO	2772	*Teleoceras*^***†***^	*hicksi*^***†***^	distal humerus	1	McKay Reservoir 1	Shutler	Hemphilian
UO	4163	*Teleoceras*^***†***^	*hicksi*^***†***^	proximal radius	1	McKay Reservoir 1	Shutler	Hemphilian
UO	9634	*Teleoceras*^***†***^	*hicksi*^***†***^	ulna	1	McKay Reservoir 1	Shutler	Hemphilian
UO	17071	*Teleoceras*^***†***^	*hicksi*^***†***^	ulna	1	McKay Reservoir 1	Shutler	Hemphilian
UO	17075	*Teleoceras*^***†***^	*hicksi*^***†***^	ulna	1	McKay Reservoir 1	Shutler	Hemphilian
UO	49287	*Teleoceras*^***†***^	*hicksi*^***†***^	distal humerus	1	McKay Reservoir 1	Shutler	Hemphilian
UCMP	113504	*Teleoceras*^***†***^	*hicksi*^***†***^	humerus	1	McKay Reservoir 1	Shutler	Hemphilian
UCMP	113507	*Teleoceras*^***†***^	*hicksi*^***†***^	tibiofibula	1	McKay Reservoir 1	Shutler	Hemphilian
UCMP	113507	*Teleoceras*^***†***^	*hicksi*^***†***^	partial ulna	1	McKay Reservoir 1	Shutler	Hemphilian
UCMP	113518	*Teleoceras*^***†***^	*hicksi*^***†***^	tibia	1	McKay Reservoir 1	Shutler	Hemphilian
UCMP	113519	*Teleoceras*^***†***^	*hicksi*^***†***^	distal humerus	1	McKay Reservoir 1	Shutler	Hemphilian
UCMP	113519	*Teleoceras*^***†***^	*hicksi*^***†***^	partial ulna	1	McKay Reservoir 1	Shutler	Hemphilian
UCMP	113519	*Teleoceras*^***†***^	*hicksi*^***†***^	proximal humerus	1	McKay Reservoir 1	Shutler	Hemphilian
UCMP	113526	*Teleoceras*^***†***^	*hicksi*^***†***^	distal humerus	1	McKay Reservoir 1	Shutler	Hemphilian
UCMP	303	*Teleoceras*^***†***^	*hicksi*^***†***^	metapodial	1	McKay Reservoir 2	Rattlesnake	Hemphilian
UCMP	306	*Teleoceras*^***†***^	*hicksi*^***†***^	metapodial	1	McKay Reservoir 3	Rattlesnake	Hemphilian
UCMP	474	*Teleoceras*^***†***^	*hicksi*^***†***^	distal metapodial	1	McKay Reservoir 4	Mascall	Barstovian
UCMP	475	*Teleoceras*^***†***^	*hicksi*^***†***^	metapodial	1	McKay Reservoir 5	Mascall	Barstovian
UCMP	477	*Teleoceras*^***†***^	*hicksi*^***†***^	metapodial	1	McKay Reservoir 6	Rattlesnake	Hemphilian
UO	5056	*Teleoceras*^***†***^	*hicksi*^***†***^	metapodial	1	McKay Reservoir 7	Shutler	Hemphilian
UO	8049	*Teleoceras*^***†***^	*hicksi*^***†***^	metapodial	1	McKay Reservoir 8	Shutler	Hemphilian
UO	8053	*Teleoceras*^***†***^	*hicksi*^***†***^	metapodial	1	McKay Reservoir 9	Shutler	Hemphilian
UO	8142	*Teleoceras*^***†***^	*hicksi*^***†***^	metapodial	1	McKay Reservoir 10	Shutler	Hemphilian
UCMP	23181	*Teleoceras*^***†***^	*hicksi*^***†***^	metacarpal 3	1	McKay Reservoir 11	Rattlesnake 11	Hemphilian
UCMP	23182	*Teleoceras*^***†***^	*hicksi*^***†***^	metatarsal 3	1	McKay Reservoir 3	Rattlesnake 11	Hemphilian
UCMP	113514	*Teleoceras*^***†***^	*hicksi*^***†***^	metapodial	1	McKay Reservoir 4	Shutler	Hemphilian
UCMP	113517	*Teleoceras*^***†***^	*hicksi*^***†***^	metapodial	1	McKay Reservoir 1	Shutler	Hemphilian
UCMP	113520	*Teleoceras*^***†***^	*hicksi*^***†***^	metapodial	1	McKay Reservoir 1	Shutler	Hemphilian
UCMP	113521	*Teleoceras*^***†***^	*hicksi*^***†***^	metapodial	1	McKay Reservoir 1	Shutler	Hemphilian
UCMP	113522	*Teleoceras*^***†***^	*hicksi*^***†***^	metapodial	1	McKay Reservoir 1	Shutler	Hemphilian
UCMP	113523	*Teleoceras*^***†***^	*hicksi*^***†***^	metapodial	1	McKay Reservoir 1	Shutler	Hemphilian
UCMP	113524	*Teleoceras*^***†***^	*hicksi*^***†***^	metapodial	1	McKay Reservoir 1	Shutler	Hemphilian
UO	10829	*Teleoceras*^***†***^	*hicksi*^***†***^	phalanx	1	McKay Reservoir 1	Shutler	Hemphilian
UCMP	113505	*Teleoceras*^***†***^	*hicksi*^***†***^	phalanx	1	McKay Reservoir 1	Shutler	Hemphilian
UCMP	113506	*Teleoceras*^***†***^	*hicksi*^***†***^	phalanx	1	McKay Reservoir 1	Shutler	Hemphilian
UCMP	113509	*Teleoceras*^***†***^	*hicksi*^***†***^	phalanx	1	McKay Reservoir 1	Shutler	Hemphilian
UO	2094	*Teleoceras*^***†***^	*hicksi*^***†***^	calcaneum	1	McKay Reservoir 1	Shutler	Hemphilian
UO	4136	*Teleoceras*^***†***^	*hicksi*^***†***^	podial	1	McKay Reservoir 1	Shutler	Hemphilian
UO	4167	*Teleoceras*^***†***^	*hicksi*^***†***^	podial	1	McKay Reservoir 1	Shutler	Hemphilian
UO	8054	*Teleoceras*^***†***^	*hicksi*^***†***^	astragalus	1	McKay Reservoir 1	Shutler	Hemphilian
UO	17063	*Teleoceras*^***†***^	*hicksi*^***†***^	podial	1	McKay Reservoir 1	Shutler	Hemphilian
UO	21886	*Teleoceras*^***†***^	*hicksi*^***†***^	astragalus	1	McKay Reservoir 1	Shutler	Hemphilian
UCMP	23178	*Teleoceras*^***†***^	*hicksi*^***†***^	lunar	1	McKay Reservoir 2	Rattlesnake 16	Hemphilian
UCMP	23179	*Teleoceras*^***†***^	*hicksi*^***†***^	calcanium	1	McKay Reservoir 3	Rattlesnake	Hemphilian
UCMP	113510	*Teleoceras*^***†***^	*hicksi*^***†***^	pisiform	1	McKay Reservoir 4	Shutler	Hemphilian
UCMP	113511	*Teleoceras*^***†***^	*hicksi*^***†***^	carpal	1	McKay Reservoir 1	Shutler	Hemphilian
UCMP	113512	*Teleoceras*^***†***^	*hicksi*^***†***^	podial	1	McKay Reservoir 1	Shutler	Hemphilian
UCMP	113513	*Teleoceras*^***†***^	*hicksi*^***†***^	podial	1	McKay Reservoir 1	Shutler	Hemphilian
UCMP	113514	*Teleoceras*^***†***^	*hicksi*^***†***^	carpal	1	McKay Reservoir 1	Shutler	Hemphilian
UCMP	113515	*Teleoceras*^***†***^	*hicksi*^***†***^	pisiform	1	McKay Reservoir 1	Shutler	Hemphilian
UCMP	113516	*Teleoceras*^***†***^	*hicksi*^***†***^	podial	1	McKay Reservoir 1	Shutler	Hemphilian
UCMP	113519	*Teleoceras*^***†***^	*hicksi*^***†***^	podial	4	McKay Reservoir 1	Shutler	Hemphilian
UCMP	113523	*Teleoceras*^***†***^	*hicksi*^***†***^	carpal	1	McKay Reservoir 1	Shutler	Hemphilian
UO	10397/ 5703	*Teleoceras*^***†***^	*hicksi*^***†***^	podial	1	McKay Reservoir 1	Shutler	Hemphilian
UCMP	113519	*Teleoceras*^***†***^	*hicksi*^***†***^	podial	3	McKay Reservoir 1	Shutler	Hemphilian
UO	G1675	*Teleoceras*^***†***^	*hicksi*^***†***^	calcaneum	1	McKay Reservoir 1	Shutler	Hemphilian
UO	G1676	*Teleoceras*^***†***^	*hicksi*^***†***^	astragalus	1	McKay Reservoir 1	Shutler	Hemphilian
UO	4184	*Teleoceras*^***†***^	*hicksi*^***†***^	axis	1	McKay Reservoir 1	Shutler	Hemphilian
UO	25504	*Teleoceras*^***†***^	*hicksi*^***†***^	axis	1	McKay Reservoir 1	Shutler	Hemphilian
UO	8055	*Teleoceras*^***†***^	*hicksi*^***†***^	NA	1	McKay Reservoir 1	Shutler	Hemphilian
AMNH	27757	*Diceros*	*bicornis*	metapodial	9	Kenya	NA	Recent
AMNH	27757	*Diceros*	*bicornis*	patella	2	Kenya	NA	Recent
AMNH	27757	*Diceros*	*bicornis*	calcaneum	1	Kenya	NA	Recent
AMNH	27757	*Diceros*	*bicornis*	podial	16	Kenya	NA	Recent
AMNH	27757	*Diceros*	*bicornis*	pisiform	1	Kenya	NA	Recent
AMNH	27757	*Diceros*	*bicornis*	phalanx	24	Kenya	NA	Recent
AMNH	27757	*Diceros*	*bicornis*	humerus	1	Kenya	NA	Recent
AMNH	27757	*Diceros*	*bicornis*	radius	1	Kenya	NA	Recent
AMNH	27757	*Diceros*	*bicornis*	ulna	2	Kenya	NA	Recent
AMNH	27757	*Diceros*	*bicornis*	scapula	1	Kenya	NA	Recent
AMNH	27757	*Diceros*	*bicornis*	femur	1	Kenya	NA	Recent
AMNH	27757	*Diceros*	*bicornis*	tibia	1	Kenya	NA	Recent
AMNH	27757	*Diceros*	*bicornis*	fibula	1	Kenya	NA	Recent
AMNH	81805	*Diceros*	*bicornis*	ulna	2	South Africa	NA	Recent
AMNH	81805	*Diceros*	*bicornis*	radius	1	South Africa	NA	Recent
AMNH	81805	*Diceros*	*bicornis*	podial	1	South Africa	NA	Recent
AMNH	81805	*Diceros*	*bicornis*	calcaneum	1	South Africa	NA	Recent
AMNH	81805	*Diceros*	*bicornis*	metapodial	1	South Africa	NA	Recent
AMNH	34739	*Diceros*	*bicornis*	scapula	1	Kenya	NA	Recent
AMNH	34740	*Diceros*	*bicornis*	tibia (juvenile)	1	Kenya	NA	Recent
AMNH	34740	*Diceros*	*bicornis*	metapodial (juvenile)	1	Kenya	NA	Recent
AMNH	34740	*Diceros*	*bicornis*	calcaneum (juvenile)	1	Kenya	NA	Recent
AMNH	14136	*Diceros*	*bicornis*	metapodial	1	NA	NA	Recent
AMNH	113779	*Diceros*	*bicornis*	femur (fetal)	1	NA	NA	Recent
AMNH	113779	*Diceros*	*bicornis*	tibia (fetal)	1	NA	NA	Recent
AMNH	113779	*Diceros*	*bicornis*	scapula (fetal)	1	NA	NA	Recent

Abbreviations: AMNH = American Museum of Natural History, UCMP = The University of California Museum of Paleontology, OU = The University of Oregon, UW = The University of Washington. A more detailed form of this table can be found in the supporting information.

Phylogenetic Data, R scripts and digital photographs associated with this study are available at Morphobank (project ID: 1238) [27] with permission from The American Museum of Natural History, The University of Washington Burke Museum, The University of Oregon Museum of Natural and Cultural History, and The University of Texas Jackson School of Geosciences Vertebrate Paleontology Laboratory. Digital photographs of fossils from the UCMP are also available through the Calphotos archive (http://calphotos.berkeley.edu/). S1 File of raw pathology scores available online through PLoS One.

### Study Species

**Fig 1:**
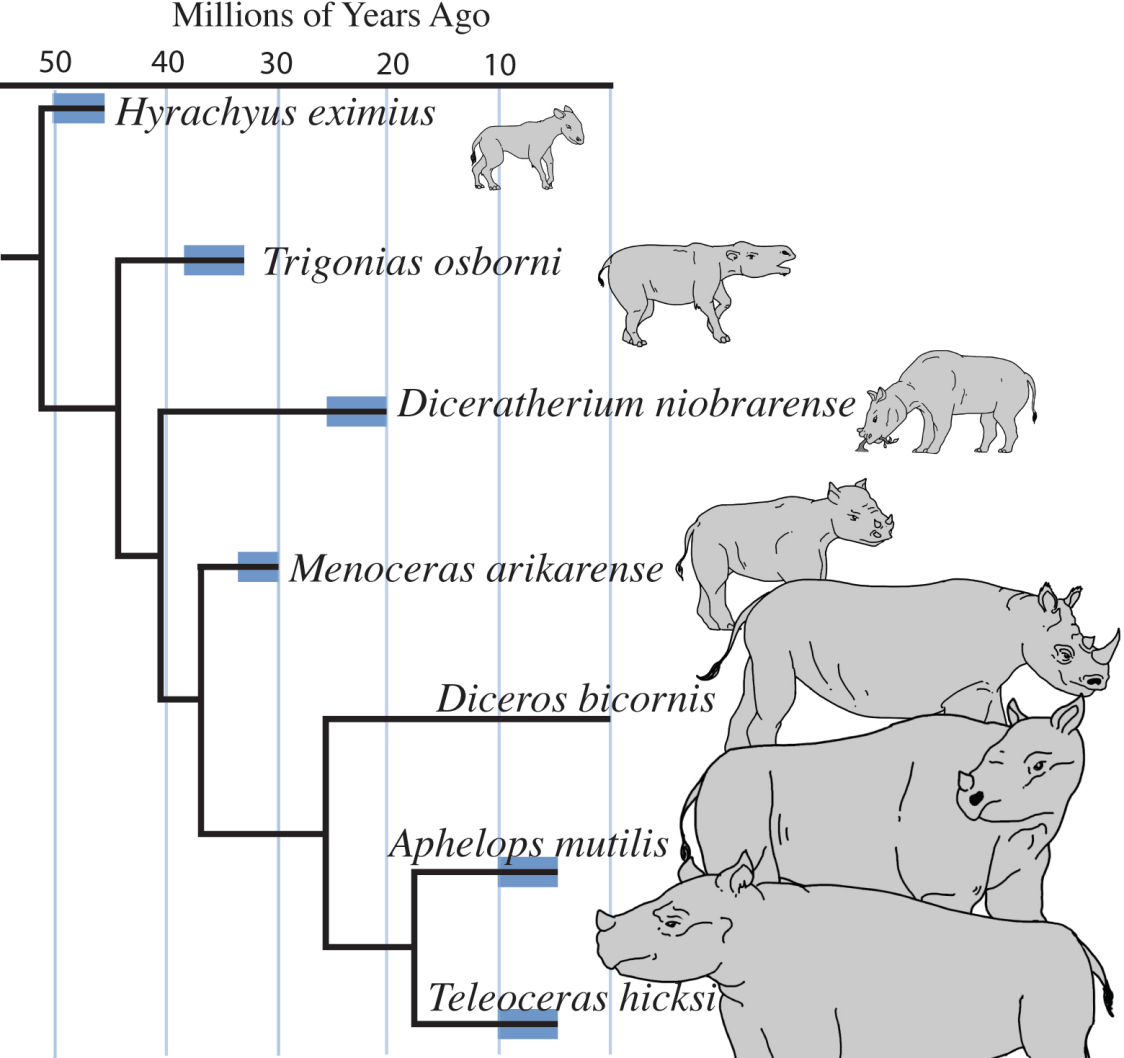
Time-calibrated phylogeny of rhinocerotid taxa used in this study with outgroup *H*. *eximius*. The thicker bars indicate the actual first and last appearance data (FAD and LAD) of the fossil localities included, not the comprehensive range of the species. *D*. *bicornis* has no blue line because only modern bones were examined. Tree was pruned from Cerdeño’s 1998 [10] morphologic phylogeny or Rhinocerotidae and time-calibrated in RStudio using the ‘equal’ setting in the function timePaleoPhy() in the software package ‘Paleotree’ [28]. Tree was set to be fully dichotomous and to extend all the way to the LAD.

*Hyrachyus eximius*, (50.5–45.4 Ma) (Fig 1) is a sister lineage of both the tapir and rhinos. This species is estimated to have weighed around 36.3 kg (equivalent in mass to a large dog), lacked horns, and was a cursorial browser [11, 23]. The rhinocerotid with the earliest first appearance datum (FAD) included in this study is the basal rhinocerotid *Trigonias osborni*. *T*. *osborni* also lacked horns and was substantially larger than *H*. *eximius* at about 677 kg [24,25]. *T*. *osborni* is known from the Chadronian (37.2 to 33.9 Ma) and was a cursorial browser.

*Menoceras arikarense* emigrated from Europe in the late Oligocene or early Miocene (24.8–20.43 Ma) and had a mass around 375 kg [24,25]. *M*. *arikarense* is notable for two firsts: horns and grazing [5]. *Diceratherium niobrarense* is larger than *M*. *arikarense* (about 1010 kg [5]). Although *D*. *niobrarense* also displays laterally paired rostral horns, it is thought be descended from *Subhyracodon* and is not considered a sister group of *M*. *arikarense* [5,18]. This rhinocerotid was present in North America in the early and middle Miocene (24.8–20.43 Ma) and was probably a browser [5]. Both *M*. *arikarense* and *D*. *niobrarense* show morphologies characteristic of increased graviportality: increased bone robusticity, more vertically-oriented pelvis [26], and widening rib cage [5]. Limb length also decreased relative to mass [5].

*Aphelops mutilis* and *Teleoceras hicksi* are similar to modern rhinos in graviportal morphology and robust limbs [5]. *A*. *mutilis* was a hornless aceratheriine browser known from the mid-Miocene to the beginning of the Pliocene (10.3–4.9 Ma) and is estimated to have weighed around 1840 kg. *T*. *hicksi* (10.3–4.9 Ma) is morphologically similar to aquatic hippos [5], but has highly hypsodont teeth [5] with enamel oxygen isotope ratios similar to terrestrial herbivores [29]. *T*. *hicksi* is estimated to have weighed around 1660 kg, is thought to have a small nasal horn and is one of the last rhinocerotids in the North American fossil record [30].

From the five modern taxa we examined in planning this study we chose *Diceros bicornis* (the black rhino) as the modern exemplar. *Diceros* (5.3332 Ma to present) weighs 800–1,350 kg [24] and is a browser with a prehensile lip specialized to grab foliage [26].

### Data Collection Procedure

Diagnosing specific diseases from osteopathologies (often the only pathologies available for study in fossil taxa) is difficult, but not impossible. Certain recognized diseases and disorders can leave distinctive features; e.g., six fingers in a human skeleton are an indicator of polydactyly. The majority of diseases display a common range of pathologies and it is these unique combinations of pathologies that are most informative. For example, irregular holes in a bone can be caused by abnormal nutrient canals, bone infection, soft-tissue swelling, or preservation damage. Arthritis may cause bones to form these irregular holes as well as bone exostoses or thinning, lipping, and fibrous, candlewax, and lumpy bone textures. Arthritis is often labeled spondylarthropathy in non-human paleopathologic studies [17,31] to acknowledge that arthritis itself is not a specific disease, but can be caused by a range of environmental, genetic, and behavioral factors depending on the system under study [16,18,19].

Each specimen was digitally photographed with a Nikon D90 camera. The camera was hand held approximately perpendicular to the photographic plane. Elongate fossils (e.g. femora or metapodials) were photographed in lateral view and fossils with irregular shapes (i.e. podials) were oriented in medial view. Proximal and distal articular surfaces were photographed as well for limb and foot elements. Vertebral elements were photographed in dorsal, ventral, proximal, and distal views. Extra photos were taken if a unique pathology was observed or for striking examples of specific pathologies.

The specimen number and corresponding photo numbers were recorded digitally and associated with a pathology index scoring and any qualitative observations (see supporting information). The presence or absence of pathology was recorded on site, while pathology severity scorings were determined from the digital photographs.

**Fig 2:**
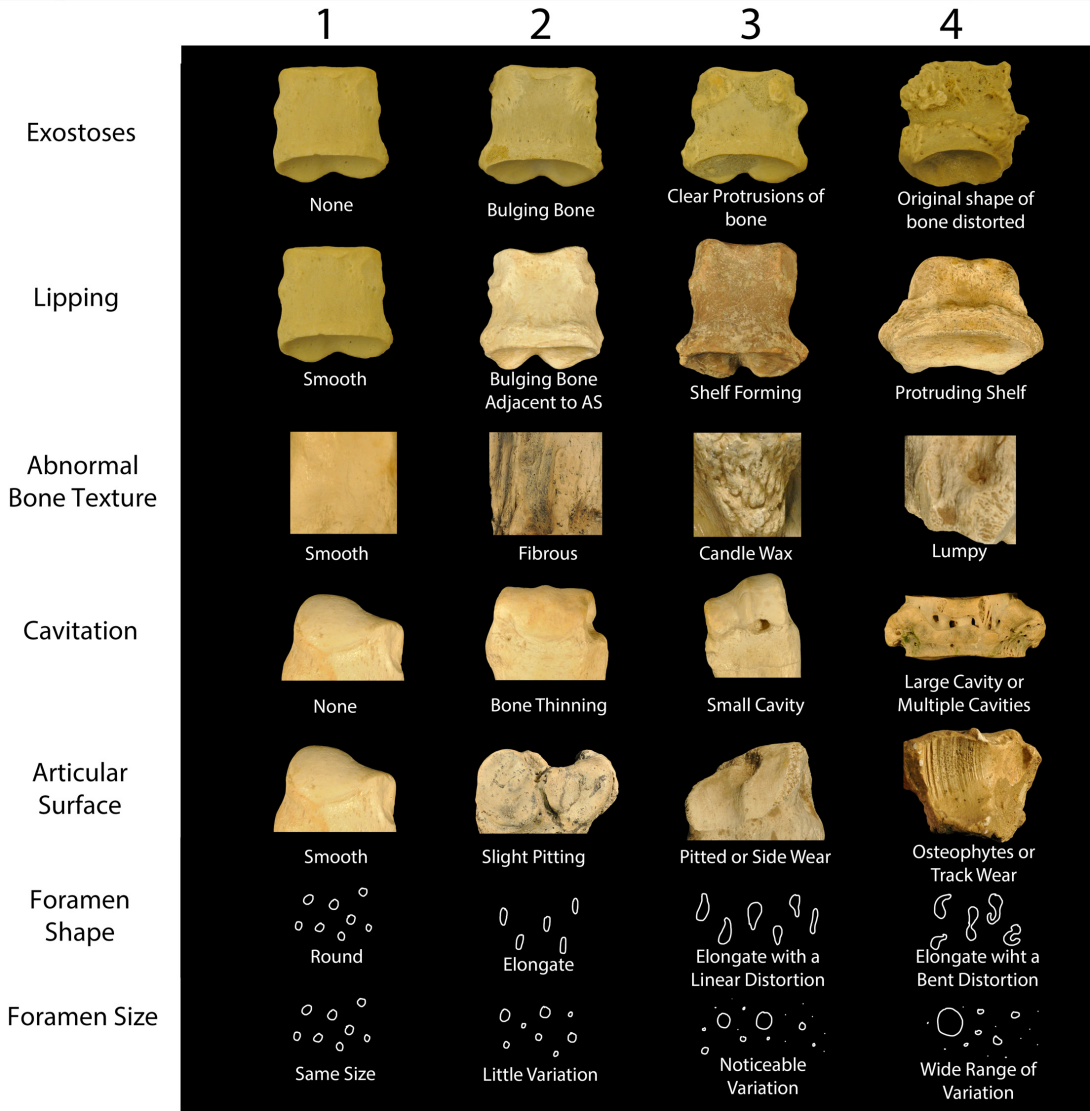
Index of Pathology (IPa) used in this study. Examples of each pathology category and the 1–4 rating system are given along with a short description.

We quantitatively described the visible surface of each bone using a category, or binning, system. We sorted our initial qualitative descriptions of possible symptoms of disease into seven different categories. The seven categories were divided into ranks from 1 (regular bone) to 4 (severe) (Fig 2). These ranks are artificial, but should allow for consistent scoring. All scoring was completed by the first author. The seven categories are exostoses, lipping, bone texture, cavitation, foramen shape, foramen size, and articular surface modification. All categories except for ‘articular surface’ refer to the nonarticular surfaces of the bones. The categories were chosen following the methodology of Aufderheide [16], Rothschild [17, 31], and Bartosiewicz et al. [21]. Analogs of this procedure have been used for decades in anthropologic [21, 22, 32] and modern cattle [21] studies. Our goal was to quantitatively describe all irregularities observed in the osteology of the Rhinocerotidae, even if they could not immediately be categorized as a pathology.

#### Category One: Exostoses

Exostoses are formations of new bone on the surface of a bone, caused by inflammation of the periosteum. Extoses appear as bumps or protuberances on an area of the bone that is expected to be smooth or relatively flat. This category includes ossification of the periosteum, ligaments, or muscle. Bones in rank one do not exhibit any exostoses. Bones in rank two show minor irregular bulging of bone. Bones in rank three show clear protrusions of irregular bone. Bones in rank four show a continuous irregular distortion of the non-articular surface of the bone.

#### Category Two: Lipping

Lipping occurs when osteophytes (commonly referred to as bone spurs) form as new bone on the margin of articular surfaces. They usually form as a series of merging osteophytes around the joint margin, but can occur singly as well. Bones in rank one do not exhibit any lipping. Bones in rank two show slight bulging of the bone adjacent to the articular surface. Bones in rank three show bulging of the bone surrounding the articular surface to the point where a prominent shelf is beginning to form. Bones in rank four show a prominent shelf adjacent to the articular surface. The shelf may be regular or irregular.

#### Category Three: Textures

Bone constantly remodels and rebuilds itself in response to localized stress. This can result in characteristic external textures. Care must be taken to not conflate exostoses (which has more to do with shape) with texture. Bones in rank one have a smooth texture. Bones in rank two have an elevated linear texture, termed fibrous. Bones in rank three have an elevated linear texture that is slightly bulging or uneven texture, likened to candle wax. Bones in rank four have an elevated, uneven, nonlinear texture.

#### Category Four: Cavitation

Cavitation is the first category concerned with loss of bone. A cavitation is a hole in the bone, usually caused by infection and/or decreased blood flow. Unlike the categories of foramen shape and size, these are relatively large areas of the bone that cannot be confused with vascularization. Bones in rank one do not exhibit any cavitation. Bones in rank two show a pockmarked appearance where the bone has lost integrity. Bones in rank three show small cavities. Bones in rank four show large cavities that may be linked together.

#### Category Five: Articular Surface

The articular surface forms the bony portion of a joint. Bones in rank one do not show any irregularities in the joint surface. Bones in rank two show a pockmarked appearance where the cartilage has been worn away. Bones in rank three show bone loss on the articular surface. Bones in rank four show eburnation of the articular surface and/or osteophyte formation.

#### Category Six: Foramen Shape

We found bone cysts can easily be confounded with vascularization (called ‘lucencies’ in Regnault et al. 2–13 [13]), so we decided to describe the degree of foramen deformation instead of labeling all foramina as cysts. Cysting (pockets or holes where localized infections occurred [16,18]) was divided into two categories (foramen shape and size). Rank one consists of circular foramina on the surface of the bone. Rank two consists of elongate or ovoid foramina. Rank three consists of elongate foramina that are irregularly ovoid, but still linear. Rank four consists of irregular, nonlinear (or bent) foramina.

#### Category Seven: Foramen Size

Rank one consists of foramina of approximately the same size. Rank two consists of foramina which show little variation in size relative to one another. Rank three consists of foramina which show moderate variation in size relative to one another. Rank four consists of foramina which show a high degrees of variation in size relative to one another.

Each bone was also classified as appendicular and axial. To explore whether there were any overt patters of regionalization in pathological expression, all appendicular elements were then divided into the functional categories: hindlimb or forelimb, and also developmental categories: girdle, stylopod, autopod, zeugopod. The overall percent expression of each category was tabulated and then compared relative to the total number of appendicular element.

### Data Analysis

Each fossil was given a score of 1–4 for each pathology, and these scores were then averaged for each taxon and pathological category, yielding 49 results. These averaged scores were then added together for each taxon (i.e. the seven pathology categories were added together for each taxon) to create an index of pathology (IPa). The minimum score possible would therefore be seven (all pathological categories in a taxon having a score of one) and the maximum would be twenty-eight (all pathological categories in a taxon having a score of four). These average scores for each taxon do not behave as ordinal data, because they are subject to the central limit of means. That is, species averages of non-continuous data behave like continuous data, especially with large sample sizes. The smallest number of specimens we analyzed for one species was 65, more than adequate to produce this effect. Consequently, we decided it was appropriate to analyze these values using continuous-data approaches: linear regression and independent contrasts.

We tested whether mass was associated with increased osteopathologic expression in two ways. First, we ran a series of linear regressions in JMP [33] with estimated mass against each individual categorical score as well as the total index. Mass estimates for extinct taxa were calculated using the total molar length (M1-3) [34] from Radinsky 1967 for *Hyrachyus eximius* [23] and Prothero 2005 [5] for all other extinct taxa, which we found to be the most reliable of available body mass estimators. Other available proxies (femur length and humerus width) produced unreasonable mass estimates [35] for one or more of the included taxa, likely as a result of the changing degree of graviportality through the history of the rhino lineage.

The second test used Felsenstein’s [36] independent contrast (IC) method to examine the influence of shared ancestry on the relationship between mass and pathology. We constructed a fully resolved tree of just the taxa in our study by paring down the results of Cerdeño 1995 [8]. The tree was time-calibrated in RStudio [37] using the packages ‘ape’ [38] and ‘paleotree’ [28] with paleotree’s function TimePaleoPhy. The r code is available in the S2 File. We used the ‘Equal’ method within TimePaleoPhy, which prevents zero-length branches, and the setting ‘add.term = TRUE’, which gave us branch lengths that took LAD into consideration. FAD and LAD for the Equal method were determined by the temporal extent of the formation at the locality where the fossils were excavated. To implement the IC method, we used the package ‘ape’ [38], to calculate the absolute values of the difference for each pair of nodes for both mass and all seven types of pathologies, as well as for the overall IPa, under a Brownian Motion model. The resulting contrasts for pathologic values were regressed against the contrast for the mass values. The r-squared and p-values for the non-phylogenetic linear regression versus the IC regression analysis were then compared.

## Results

Overall, geologically older taxa show the smallest relative abundance of pathologic elements, while the greatest pathologic expression was seen in the more derived taxa, which were also the most massive taxa sampled in North America. The one exception is the extant species, *D*. *bicornis*, which appears later, yet is less massive and less pathologic overall than *A*. *mutilis* and *T*. *hicksi*.

When taxa are considered separately, *H*. *eximius* displayed low osteopathologic expression (~28%), most of which was expressed as cysting and exostoses in the podials. *T*. *osborni* and *D*. *niobrarense* also displayed a greater degree of pathologic expression in the distal elements. The *M*. *arikarense* fossil assemblage displayed prominent exostoses. In smaller elements (i.e. podials) the non-articular surfaces would be almost entirely composed of exostoses. *A*. *mutilis* and *T*. *hicksi* commonly contained large visible cysts and rank three, candlewax, bone texture. Only two fossils displayed eburnation, in *A*. *mutilis* on the articular surfaces of a proximal tibia (UCMP F-30266) and in *T*. *hicksi* on a distal humerus (UO F-2772). *A*. *mutilis* also had the highest percent expression of any one pathology (in this case, foramen shape), while *D*. *bicornis* had comparatively more foramen variation adjacent to the articular surfaces and fewer exostoses than the other robust taxa. One specimen (VPL M-8259) had flat ‘rice grain’ crystals on the proximal articular surface of right radius and ulna, as well as the distal articular surface of the humerus, a possible indication of gout [16, 18]. Tendon ossification was only seen in *A*. *mutilis* and *T*. *hicksi*. Of note, most of the articular surfaces of the synovial joints in both extinct and extant taxa appeared smooth and free of damage.

Overall index of pathology (IPa) scores were between 8 and 18, Table 3. The two oldest lineages, *H*. *eximius* and *T*. *osborni*, had an overall pathologic score of 8.8 and 11.06 respectively. The next oldest lineage, *D*. *niobrarense*, had an overall score of 12.81, while *M*. *arikarense* had an overall score of 13.31. *T*. *hicksi* had an overall score of 14.26, while *A*. *mutilis* had an overall score of 17.57. The modern rhino, *D*. *bicornis*, had an overall IPa of 12.23.

**Table 3:** Frequency of Pathology Scores and IPa grouped by Osteopathologies per Taxa.

		Exostoses	Lipping	Abnormal Bone Texture	Cavitation	Foramen Shape	Foramen Size	Articular Surface	Overall
*H*. *eximius*^*†*^	1	261	345	256	307	277	158	579	2183
	2	127	46	125	81	102	292	16	789
	3	3	0	9	4	13	15	1	45
	4	0	0	0	0	0	0	0	0
	**IPa**	**1.35**	**1.11**	**1.36**	**1.19**	**1.32**	**1.40**	**1.07**	**8.80**
*T*. *osborni*^*†*^	1	19	78	17	86	56	52	95	403
	2	88	34	81	17	50	42	16	328
	3	7	2	15	11	8	20	3	66
	4	0	0	1	0	0	0	0	1
	**IPa**	**1.90**	**1.33**	**2**	**1.34**	**1.58**	**1.72**	**1.19**	**11.06**
*M*. *arikarense*^*†*^	1	12	59	9	45	21	10	65	221
	2	58	32	59	21	43	26	21	260
	3	21	1	23	26	26	53	6	156
	4	1	0	1	0	2	3	0	7
	**IPa**	**2.18**	**1.37**	**2.16**	**1.77**	**2.01**	**2.51**	**1.31**	**13.31**
*D*. *niobrarense*^*†*^	1	14	33	3	36	25	26	52	200
	2	41	36	45	24	33	29	20	245
	3	19	6	23	14	17	20	3	122
	4	1	0	4	1	0	0	0	33
	**IPa**	**2.10**	**1.61**	**2.38**	**1.69**	**1.85**	**1.88**	**1.31**	**12.81**
*A*. *mutilis*^*†*^	1	0	61	10	35	3	0	73	182
	2	47	49	44	19	25	12	39	235
	3	73	20	53	44	81	74	10	355
	4	11	1	23	33	22	45	1	136
	**IPa**	**2.69**	**1.75**	**2.96**	**2.54**	**2.86**	**3.26**	**1.53**	**17.57**
*T*. *hicksi*^*†*^	1	12	54	9	27	9	9	70	190
	2	34	26	34	36	33	20	10	193
	3	31	2	37	18	38	46	2	174
	4	5	0	2	1	2	7	0	17
	**IPa**	**2.49**	**1.37**	**2.43**	**1.82**	**2.4**	**2.59**	**1.169**	**14.26**
*D*. *bicornis*	1	16	47	9	62	14	7	68	298
	2	56	26	46	13	37	33	7	218
	3	3	2	14	0	24	35	0	78
	4	0	0	6	0	0	0	0	6
	**IPa**	**1.83**	**1.4**	**2.23**	**1.17**	**2.13**	**2.37**	**1.09**	**12.23**

IPa = Index of Pathology, the average of all pathology scores (1 through 4) for all the individuals of a given taxa. Overall IPa is the sum of the seven individual averages. Frequency is unbolded, IPa is bolded.

When we regressed mass for each taxon against each of the seven osteopathology categories Table 4, four of the pathologies (exostoses, abnormal textures, foramen shape, and foramen size variation) had p-values less than or equal to 0.05. A linear correlation of mass against the overall pathologic scores was found to be significant (p = 0.04) and accounted for about 52% of the variation (r^2^ adj.). The IC analysis comparing mass and the seven osteopathology categories was also significant (p ≤ 0.05) for the both foramen shape and the overall pathologic index, with mass accounting for 42% of the overall variation.

**Table 4:** Linear Regression and Independent Contrast Regression against Mass.

		Overall Index	Exostoses	Lipping	Abnormal Bone Texture	Cavitation	Foramen Shape	Foramen Size	Articular Surface
**Linear Contrasts**	R^2^ Adj	0.5199	0.4772	0.2389	0.659	0.3325	0.6421	0.4797	-0.11
	F	7.498	6.477	2.884	12.6	3.988	11.76	6.532	0.4056
	P	0.04087	0.05158	0.1502	0.0164	0.1023	0.01864	0.05091	0.5522
**Independent Contrasts**	R^2^ Adj	0.4235	0.4011	-0.01015	0.5236	0.2023	0.5263	0.3932	-0.1356
	F	5.408	5.018	0.9397	70595	2.522	7.666	4.888	0.2836
	P	0.03759	0.07522	0.3769	0.04003	0.1731	0.03942	0.07802	0.6171

Linear Regression and Independent Contrast Regression Statistics against Mass. R^2^-adjusted, F Statistic and P values for linear and independent contrast (IC) regressions for each category of pathology are included.

**Fig 3:**
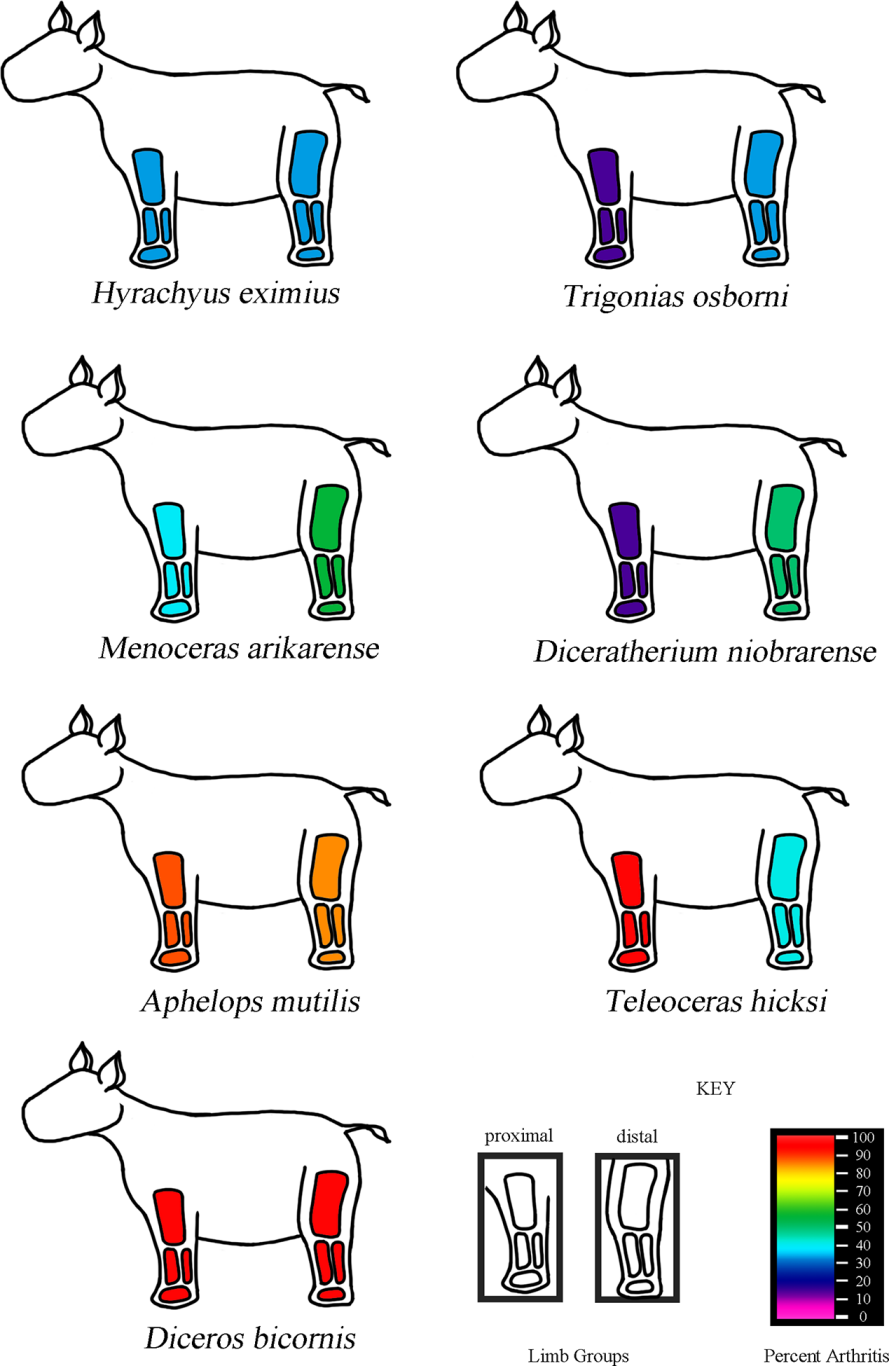
Comparison of the forelimb and hindlimb. A color spectrum is used to indicate the percent of elements displaying any osteopathology in the forelimb and hindlimb, respectively. The closer to the red portion of the color spectrum, the higher percentage. The closer to the violet portion of the color spectrum, the lower the percentage. Rhino figures do not display relative size.

**Fig 4:**
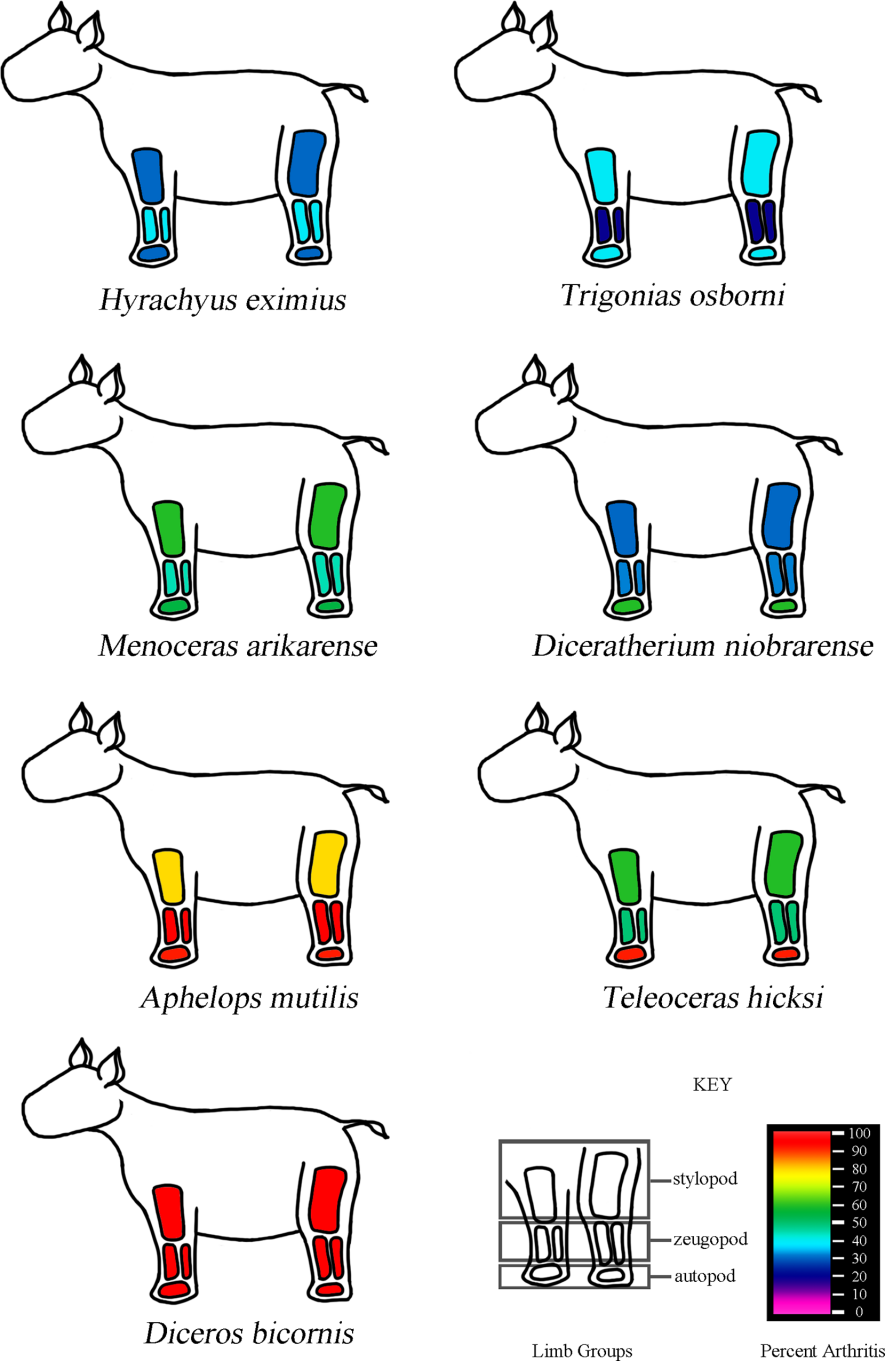
Comparison of limb regions. A color spectrum is used to indicate the percent of elements displaying any osteopathology in the stylopod, zeugopod, and autopod regions, respectively. The closer to the red portion of the color spectrum, the higher percentage. The closer to the violet portion of the color spectrum, the lower the percentage. Rhino figures do not display relative size.

We were also interested in testing whether certain bones or regions of the appendicular skeleton (which comprises the majority of the data) displayed a greater amount of pathology than other bones or regions of the appendicular skeleton. We divided all appendicular elements into the functional categories: hindlimb or forelimb (Fig 3), and also developmental categories: girdle, stylopod, zeugopod, autopod (Fig 4). For example, if the stresses generating the osteopathology were greater in the distal parts of the limb, one might expect greater pathology in the autopod (manus and pes) than the stylopod (humerus and femur). We found no significant difference in pathological expression between different regions of the appendicular skeleton.

## Discussion

In our study we found that mass can explain roughly 50% of the osteopathological expression. *A*. *mutilis*, surprisingly, had the highest pathology scores by a wide margin, while *T*. *hicksi*, which was close to *A*. *mutilis* in estimated mass, had scores similar to the smaller *D*. *niobrarense* and *M*. *arikarense*. Both the overall expression of pathology and the subcategory of foramen shape were significant when regressed against mass regardless of whether phylogeny was taken into account or not. However, the r^2^ value in the vicinity of 0.5 suggests that other factors besides mass, such as bone robusticity, cursoriality or environment, could play a significant role in pathological expression. There might be a tradeoff between a lineage increasing in size or weight and abnormal textures, lipping, and other pathologies that intuitively should be selected against on an evolutionary scale. Lower levels of expression (categories 1 and 2) were more common, but no taxon was entirely pathology-free. The ‘maximum operational level’ of different pathologies may be similar or drastically different in different vertebrate lineages, which in turn could lead to diverse selection pressures.

Longevity could also be a factor in pathological expression. There is a positive correlation in the Mammalia between body mass and lifespan, although there is a great amount of variation [39]. The larger taxa may be living longer, which could increase the likelihood of osteoarthritis, synovitis, traumatic injury, etc. Captive mammals that live longer than their wild counterparts often display these pathologies [4, 13, 14, 15]. Pathology could be a reflection of ontogeny. However, a longer lifespan does not necessarily increase only the geriatric portion of a mammal’s life. In an animal with a longer lifespan bone would presumably stay healthy for the same proportion of a mammal’s life as the shorter-lived counterpart, but this remains to be tested [40]

Our main difficulty in this study was to establish a measurement method for pathology. In anthropology several qualitative and quantitative metrics have been used to study paleopathology [16, 18, 19]; paleontology also has no universal methodology for identifying and analyzing paleopathologies, but several parallel methods [12, 13, 14, 17, 21, 27]. Our method uses a scoring system that is focused on parsing out the severity of symptoms, not direct diagnoses of disease. It is possible to apply these separate pathology categories to studies across the vertebrate kingdom. Comparison of pathological expression between these vastly different taxa could lead to new insights into bone repair, species and lineage-level responses to pathology, and the uniformity of bone-related diseases over time.

Synovitis, not arthritis, may be the proximal cause of the pathologies observed. Notably, we found that most of the pathology in the taxa we studied was located immediately adjacent to the articular surface of joints and not in the articular surface itself. That is, the articular surface itself appeared healthy (that is, not scarred or pitted) in all but five individual specimens even in individuals with advanced exostoses and abnormal bone textures. This could indicate that the joints (and therefore the organism) are functional well after pathologies begin to appear and swelling of the synovium caused the observed cortical erosion [16].

The overall picture painted by our results shows a measureable increase in the percentage of elements that display osteopathologies related to arthritis from the older to newer branches in the North American rhinocerotid lineage, consistent with earlier observations [4, 13, 14, 15, 16, 17]. Our initial hypothesis was that more massive rhino species would display a greater frequency of osteopathologies as increased loading pressure on the limbs caused microfractures that resulted in inflammation and abnormal bone textures. With our current sample, we found a significant correlation of pathology with mass, suggesting that increasing size in the lineage was partially responsible for osteopathologies. This study is not powerful enough to conclude if these pathologies are related primarily to arthritis or is a multifactorial response or a range of diseases. Because size increase is a common adaptation in terrestrial herbivores for both eating low-quality diets [26, 41, 42] and resisting predation pressure in open environments [26, 43], it seems that adaptations for food and predator avoidance may incur a cost in bone stress and osteopathology. The accumulation of pathologies in a lineage may no longer be solely a herald of disaster, but of adaptation as well.

## Supporting Information

S1 File Raw pathology Scores for all Rhinocerotidae specimens used in this study. Picture numbers correspond to digital photographs uploaded to Morphobank (project ID: 1238) [27]. Information available with permission from The American Museum of Natural History, The University of Washington Burke Museum, The University of Oregon Museum of Natural and Cultural History, and The University of Texas Jackson School of Geosciences Vertebrate Paleontology Laboratory.
(XLSX)

S2 File R Code and Required Files for Analysis. R Code can be run in RStudio [37] using the packages ‘ape’ [38] and ‘paleotree’ [28] with paleotree’s function TimePaleoPhy. Trees from Cerdeño (1995) [8].
(ZIP)
