# Correction: Osteopathology in Rhinocerotidae from 50 Million Years to the Present

**DOI:** 10.1371/journal.pone.0160793

**Published:** 2016-08-02

**Authors:** Kelsey T. Stilson, Samantha S. B. Hopkins, Edward Byrd Davis

After publication, it came to the authors’ attention that the *Diceratherium* specimens from the John Day localities used in this study are now recognized as two species, *Diceratherium armatum* and *Diceratherium annectens*. *Diceratherium niobrarense* is still applicable to other *Diceratherium* populations during this time period, but is no longer considered appropriate for the John Day specimens.
